# Transcriptome-enabled discovery and functional characterization of enzymes related to (*2S*)-pinocembrin biosynthesis from *Ornithogalum caudatum* and their application for metabolic engineering

**DOI:** 10.1186/s12934-016-0424-8

**Published:** 2016-02-04

**Authors:** Lei Guo, Xi Chen, Li-Na Li, Wei Tang, Yi-Ting Pan, Jian-Qiang Kong

**Affiliations:** Institute of Materia Medica, Chinese Academy of Medical Sciences and Peking Union Medical College (State Key Laboratory of Bioactive Substance and Function of Natural Medicines & Ministry of Health Key Laboratory of Biosynthesis of Natural Products), Beijing, 100050 China; School of Medicine of Wuhan University, Wuhan, China; School of Chemical Engineering, Beijing Institute of Petrochemical Technology, Beijing, China

**Keywords:** (*2S*)-Pinocembrin, *Ornithogalum caudatum*, Metabolic engineering, 4-Coumarate:coenzyme A ligase, Chalcone synthase, Chalcone isomerase

## Abstract

**Background:**

(*2S*)-Pinocembrin is a chiral flavanone with versatile pharmacological and biological activities. Its health-promoting effects have spurred on research effects on the microbial production of (*2S*)-pinocembrin. However, an often-overlooked salient feature in the analysis of microbial (*2S*)-pinocembrin is its chirality.

**Results:**

Here, we presented a full characterization of absolute configuration of microbial (*2S*)-pinocembrin from engineered *Escherichia coli*. Specifically, a transcriptome-wide search for genes related to (*2S*)-pinocembrin biosynthesis from *Ornithogalum caudatum*, a plant rich in flavonoids, was first performed in the present study. A total of 104,180 unigenes were finally generated with an average length of 520 bp. The Kyoto Encyclopedia of Genes and Genomes (KEGG) pathway mapping assigned 26 unigenes, representing three enzyme families of 4-coumarate:coenzyme A ligase (4CL), chalcone synthase (CHS) and chalcone isomerase(CHI), onto (*2S*)-pinocembrin biosynthetic pathway. A total of seven, three and one full-length candidates encoding 4CL, CHS and CHI were then verified by reverse transcription polymerase chain reaction, respectively. These candidates were screened by functional expression in *E. coli* individual or coupled multienzyme reaction systems based on metabolic engineering processes. *Oc4CL1*, *OcCHS2* and *OcCHI* were identified to be *bona fide* genes encoding respective pathway enzymes of (*2S*)-pinocembrin biosynthesis. Then *Oc4CL1*, *OcCHS2* and *MsCHI* from *Medicago sativa*, assembled as artificial gene clusters in different organizations, were used for fermentation production of (*2S*)-pinocembrin in *E. coli*. The absolute configuration of the resulting microbial pinocembrin at C-2 was assigned to be *2S*-configured by combination of retention time, UV spectrum, LC–MS, NMR, optical rotation and circular dichroism spectroscopy. Improvement of (*2S*)-pinocembrin titres was then achieved by optimization of gene organizations, using of codon-optimized pathway enzymes and addition of cerulenin for increasing intracellular malonyl CoA pools. Overall, the optimized strain can produce (*2S*)-pinocembrin of 36.92 ± 4.1 mg/L.

**Conclusions:**

High titre of (*2S*)-pinocembrin can be obtained from engineered *E. coli* by an efficient method. The fermentative production of microbial (*2S*)-pinocembrin in *E. coli* paved the way for yield improvement and further pharmacological testing.

**Electronic supplementary material:**

The online version of this article (doi:10.1186/s12934-016-0424-8) contains supplementary material, which is available to authorized users.

## Background

**Fig. 1:**
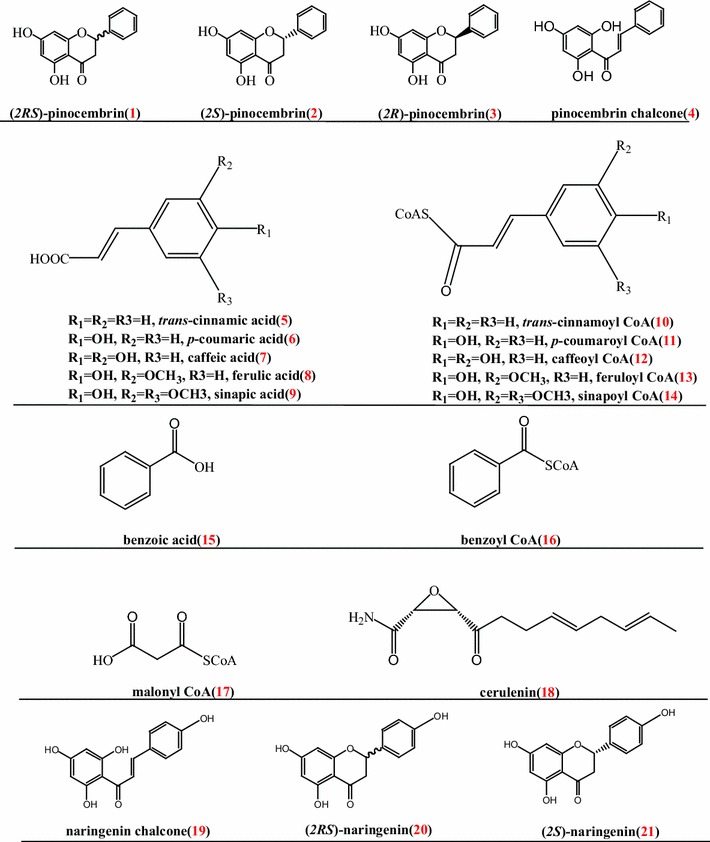
Chemical structures of compounds investigated in this study

**Fig. 2:**
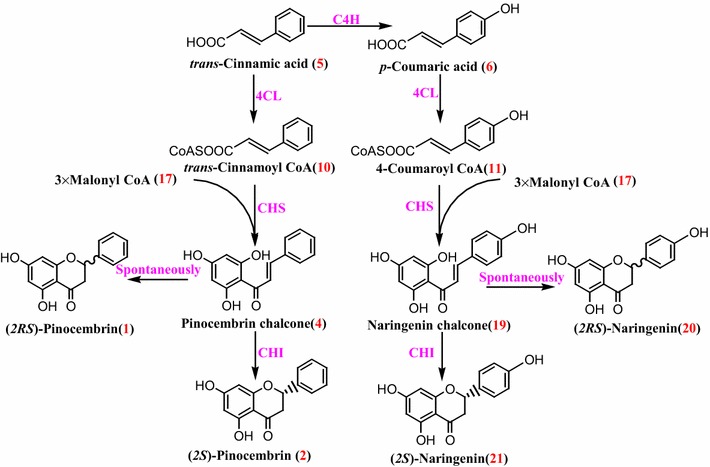
Biosynthetic pathway of (*2S*)-pinocembrin (**2**)

Pinocembrin (**1**, Fig. 1), also named 5, 7-dihydroxyflavanone or dihydrochrysin, is a kind of chiral flavonoid made up of two enantiomer forms, (*2S*)-pinocembrin (**2**, Fig. 1) and (*2R*)-pinocembrin (**3**, Fig. 1). Unlike infrequent (*2R*)-pinocembrin (**3**) [1, 2], (*2S*)-pinocembrin (**2**) was shown to widely occur in most of the propolises [3–5] and plants, like *Cryptocarya chingii* [6], *Quinchamalium majus* Brong [7] and *Glycyrrhiza glabra* [8]. (*2S*)-pinocembrin (**2**) exerts versatile pharmacological and biological activities including antimicrobial properties [5, 9, 10], anticancer activity [10, 11], anti-inflammatory effect [9, 10] and antioxidant action [9, 10], which makes it a promising compound with pharmaceutical potential. The racemic pinocembrin (**1**) had been therefore approved to enter phase II clinical trials as a potential therapeutic for stroke by the State Food and Drug Administration of China [12].

(*2S*)-pinocembrin (**2**) is obtained by extracting from plants [6–8] or propolis [4, 11], chemical synthesis [13–16] and microbial production by metabolic engineering or synthetic biology [17–20]. Although plants or propolis are still a primary source of (*2S*)-pinocembrin (**2**) [21–24], the production of plant- or propolis-derived (*2S*)-pinocembrin (**2**) is hampered by the low availability and complicated purification procedures. Apart from natural sources, it has been noted that (*2S*)-pinocembrin (**2**) can be chemically synthesized. However, chemical synthesis faces several obstacles such as the usage of toxic chemicals, extreme reaction conditions and sophisticated enantiomeric resolution [13, 14, 16, 25]. In response to the poor yield of extraction from natural sources and poor chemical synthesis efficiency, research groups have directed their attention to the microbial production of (*2S*)-pinocembrin (**2**) [18–20]. This approach expresses the biosynthetic pathway of (*2S*)-pinocembrin (**2**) in many more amenable heterologous hosts to improve pinocembrin yields with a more economical and environment-friendly manner.

The biosynthesis of (*2S*)-pinocembrin (**2**) begins with the phenylpropanoid pathway, in which *trans*-cinnamic acid (**5**, *t*-CA) is used to generate *trans*-cinnamoyl CoA **(10)** by 4-coumarate:coenzyme A ligase(4CL). Chalcone synthase (CHS) catalyzes the stepwise condensation of three acetate units from malonyl CoA (**17**) with *trans*-cinnamoyl CoA **(10)** to yield pinocembrin chalcone (**4**). The latter is then converted to (*2S*)-pinocembrin (**2**) by the action of chalcone isomerase (CHI) in vivo or to racemic pinocembrin non-enzymatically (Fig. 2). The health-promoting effects of (*2S*)-pinocembrin (**2**) have spurred on research efforts towards the development of microbial production platforms using phenylpropanoid and flavonoid biosynthetic enzymes [18, 26–30]. Up to date, pinocembrin has been obtained from engineered *Escherichia coli* [18, 20, 31], *Saccharomyces cerevisiae* [29, 30] and *Streptomyces venezuelae* [27] by combinational expression of pathway enzymes with diverse genetic sources. These studies, although valuable, have a distinct defect, namely no full characterization of stereochemistry of microbial (*2S*)-pinocembrin (**2**). Besides this, it will be necessary to test much more structural genes coming from varied origins because the cloning and the characterization of diverse genes can offer new perspectives in the development of recombinant microorganisms capable of a high and optimized production of microbial (*2S*)-pinocembrin (**2**). With these in mind, this study describes the isolation and functional expression of enzymes related to a complete (*2S*)-pinocembrin (**2**) pathway from *Ornithogalum caudatum* for the first time. Importantly, these enzymes were then used to successfully rebuild a biosynthetic circuit in *E. coli* to acquire (*2S*)-pinocembrin (**2**), which broadened the genetic sources of gene parts used for microbial (*2S*)-pinocembrin (**2**) production. What’s more, the present study fully characterized the absolute configuration of microbial (*2S*)-pinocembrin (**2**), which is uniquely value for yield improvement and further pharmacological testing of chiral (*2S*)-pinocembrin (**2**).

## Results

### KEGG pathway analysis of *O. caudatum* unigenes

The transcriptome is the universe of expressed transcripts within a cell at some particular state. Transcriptome sequencing is a high-throughput approach and can yield a tremendous amount of sequences in each run, far greater than that produced by traditional techniques. Transcriptome sequencing, therefore, can greatly accelerate full-length genes isolation. In the present study, a total of 104,180 unigenes with an average length of 520 bp were acquired from transcriptome *de novo* assembly. These unigene sequences were aligned to KEGG pathways by KEGG analysis. Results showed varied unigenes were assigned to every step of (*2S*)-pinocembrin (**2**) biosynthesis (Additional file 1: Table S1). Totally, 19, 3 and 4 unigenes showing high similarity with 4CL, CHS and CHI were retrieved from transcriptome sequence, respectively (Additional file 1: Table S1). These unigenes were further analyzed by BLAST X for their ORF (open reading frame) identification. Some of these unigenes were predicted to contain full-length complementary DNA (cDNA) sequences and the others had partial cDNA encoding sequences.

These predicted full-length cDNA sequences can be isolated from *O. caudatum* cDNA directly by nested polymerase chain reaction (PCR). The missing sequences of these tentatively partial cDNA, however, were obtained mainly by RACE (rapid amplification of cDNA end) [32]. Finally, a total of 11 full-length cDNAs, including seven 4CL-like sequences, three CHS-like cDNA and one full-length CHI-like fragment, were isolated from *O. caudatum* (Additional file 1: Table S1). All of these ORFs were then inserted to the cloning vector pEASY™-T1 Simple vector for sequencing. The results verified that these cDNA sequences were identical with the result from transcriptome sequencing, which means the real genes *in planta*. Therefore, these sequences were deposited in the GenBank database (Table 1).

**Table 1:** Full-length cDNAs related to (*2S*)-pinocembrin (**2**) biosynthesis

Gene	nt (bp)	Polypeptide (aa)	Accession number
*Oc4CL1*	1638	545	KF241990.1
*Oc4CL2*	1644	547	KM393177.1
*Oc4CL3*	1566	521	KM393178.1
*Oc4CL4*	1710	569	KM393179.1
*Oc4CL5*	1644	547	KM393180.1
*Oc4CL6*	1695	564	KM393181.1
*Oc4CL7*	1623	540	KM393182.1
*OcCHS1*	1149	382	KM393183.1
*OcCHS2*	1173	390	KM393184.1
*OcCHS3*	1152	383	KM393185.1
*OcCHI*	633	210	KR822738.1

### cDNA isolation and functional characterization of 4CL gene family

A 4CL gene family harboring seven full-length cDNAs, namely Oc4CL1-7, was isolated from *O. caudatum* by nested PCR (Table 1). These cDNAs were cloned into pEASY™-T1 to generate pEASY-Oc4CLs for sequencing. After sequences verification, the *Oc4CL* genes were cloned into *E. coli* vector pET-28a (+) resulting in recombinant vectors pET28a-Oc4CLs for heterologous expression by In-Fusion^®^ method, respectively.

Various pET28a-Oc4CLs were transformed into *E. coliTrans*etta (DE3) to acquire engineered *E. coli* [pET28a-Oc4CLs] for heterologous expression of Oc4CLs. Both SDS–PAGE (sodium dodecyl sulfate polyacrylamide gel electrophoresis, Additional file 2: Fig. S1) and Western-blot (Additional file 3: Fig. S2) results demonstrated that there were indeed Oc4CL proteins expressed in the *E. coli* culture.

**Fig. 3:**
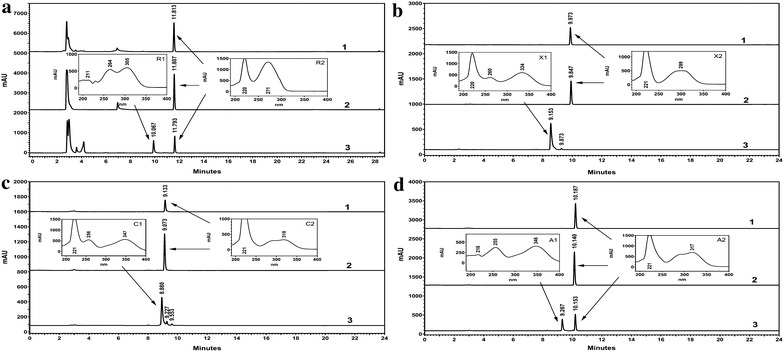
HPLC analysis of reaction products from *E. coli* [pET28a] (*1*), heat denatured protein extracts derived from *E. coli* [pET28a-Oc4CL1] (*2*) and crude protein extracts of *E. coli* [pET28a-Oc4CL1] (*3*) using *trans*-cinnamic acid (**a**), *ρ*-coumaric acid (**b**), caffeic acid (**c**), and ferulic acid (**d**) as the substrates. R2, X2, C2, A2, R1, X1, C1 and A1 refer to substrates *trans*-cinnamic acid (**5**), *ρ*-coumaric acid (**6**), caffeic acid (**7**), ferulic acid (**8**), and their corresponding products *trans*-cinnamoyl CoA (**10**), 4-coumaroyl CoA (**11**), caffeoyl CoA (**12**) and feruloyl CoA (**13**), respectively. Detection was set at 270 nm for reaction product of *trans*-cinnamic acid (**5**), 320 nm for enzymatic products of *ρ*-coumaric acid (**6**), caffeic acid (**7**) and ferulic acid (**8**). *mAU* Milliabsorbance units

Following induction of transformed *E. coli* cells with isopropyl-β-d-thiogalactopyranoside (IPTG), the crude extracts of *E. coli* [pET28a-Oc4CLs] were used to perform enzyme assays using six possible substrates, *viz*., *trans*-cinnamic (**5**), *p*-coumaric (**6**), caffeic (**7**), ferulic (**8**), sinapic (**9**) and benzoic (**15**) acids, respectively. High-performance liquid chromatography-diode array detector (HPLC-DAD) results showed only Oc4CL1 has reactions with substrates **5**–**8** (Fig. 3). The UV-Vis spectras of these products were identical with standard compounds reported early [33–36]. No products, however, were found in the reaction system with compounds **9** or **15** as a substrate (Data not shown). LC–MS analyses of compounds **5**–**8**, namely substrates R2, X2, C2 and A2 and their corresponding products R1, X1, C1 and A1 displayed their [M-H]^−^ ions at *m/z* 148.1, 164.1, 180.0, 194.1, 896.2, 912.2, 928.2 and 942.2, corresponding to the calculated mass for *trans*-cinnamic acid (**5**), *p*-coumaric acid (**6**), caffeic acid (**7**), ferulic acid (**8**) and their corresponding CoA thioesters. To study further the structure of products R1, X1, C1 and A1, 8 mg purified products each were produced by HPLC and applied to NMR (Table 2). The ^1^H NMR spectrum of R1 showed the signals of following protons: one set of A_2_B_2_X type aromatic protons at δ 7.37 (3H, m, H-27, H-28, H-30), 7.43 (2H, dd, *J* = 1.5, 8.0 Hz, H-3′, H-5′), and 7.49 (2H, d, *J* = 7.8 Hz, H-2′, H-6′), as well as a pair of *trans*-coupled olefinic proton signals at δ 6.66 (1H, d, *J* = 16.0 Hz, H-23) and 7.33 (1H, d, *J* = 16.0 Hz, H-24) ascribable to the styrene moiety; two independent aromatic protons at δ 8.01 (1H, s, H-2), and 8.31 (1H, s, H-5), as well as proton signals for a ribofuranose at δ 5.99 (1H, d, *J* = 6.0 Hz, H-6), 4.70 (1H, br m, H-7), 4.67 (1H, br m, H-8), 4.48 (1H, br m, H-9), and 4.18 (2H, br m, H-10) attributable to the adenosine unit. In addition, **R1** showed a series of methylene protons at δ 3.49 (1H, m, H-11a), 3.77 (1H, m, H-11b), 3.39 (2H, m, H-17), 2.39 (2H, m, H-18), 3.34 (2H, m, H-20) and 3.05 (2H, m, H-21), as well as a methine proton at δ 3.95 (1H, m, H-15) assignable to the side chain of coenzyme A. The ^13^C NMR spectrum presented signals of two carbonyl at δ 177.6 (C-16), 176.9 (C-19), as well as five aromatic carbons at δ 158.3 (C-1), 155.6 (C-2), 152.0 (C-3), 121.4 (C-4), and 142.4 (C-5), which were in agreement with the coenzyme A unit. The ^13^C NMR spectrum also displayed carbon signals assignable to a cinnamoyl group at δ 196.1 (C-22), 127.0 (C-23), 144.5 (C-24), 133.9 (C-25), 131.3 (C-26, C-30), 131.9 (C-27, C-29) and 136.3 (C-30). Based on the above observations, compound **R1** was assigned as *trans*-cinnamoyl-CoA (**10**). Careful analyses of NMR (^1^H, ^13^C) spectra revealed the structure of **X1** was similar to that of **R1**, except that the cinnamoyl group was replaced by a *p*-coumaroyl group in **X1**. The ^1^H NMR spectrum of **X1** displayed one set of A_2_B_2_ type aromatic protons at δ 7.22 (2H, dd, *J* = 1.5, 9.0 Hz, H-26, H-30), 6.70 (2H, dd, *J* = 1.5, 9.0 Hz, H-27, H-29) attributable to the *p*-hydroxybenzoyl unit. The ^13^C NMR spectrum of **X1** also exhibited carbon signals at δ 128.5 (C-25), 118.7 (C-26, C-30), 133.5 (C-27, C-29), and 161.3 (C-28) assignable to a *p*-hydroxybenzoyl moiety. Thus compound **X1** was elucidated as 4-coumaroyl CoA (**11**). By analysis of the NMR (^1^H, ^13^C) spectroscopic data, compound **C1** was found to be identical with **R1** except for the difference of benzene ring of the styrene moiety. The ^1^H NMR spectrum of **C1** exhibited one set of ABX aromatic protons which were ascribable to a 3, 4 – dihydroxyphenyl moiety at δ 6.83 (1H, br s, H-26), 6.74 (1H, d, *J* = 8.5 Hz, H-29), and 6.79 (1H, dd, *J* = 1.5, 8.5 Hz, H-30). The ^13^C NMR spectrum of **C1** displayed carbon signals for caffeoyl group at δ195.7 (C-22), 124.3 (C-23), 144.7 (C-24), 128.9 (C-25), 113.7 (C-26), 150.9 (C-27), 150.2 (C-28), 118.3 (C-29), and 126.5 (C-30). On the basis of these observation, compound **C1** was assigned as caffeoyl-CoA (**12**). A comparsion of the NMR (^1^H, ^13^C) spectral data of **C1** with those of **A1** indicated the latter differed from **C1** only in the moiety at C-27 of the caffeoyl moiety. Instead of a caffeoyl unit of **C1**, a methoxyl was connected to the C-27 and a feruloyl group was present in **A1**. According to the results of ^1^H NMR and ^13^C NMR spectral data, compound **A1** was elucidated as feruloyl-CoA (**13**).

**Table 2:** ^1^H and ^13^C NMR data for compounds R1, X1, C1 and A1 (500 MHz for ^1^H NMR and 125 MHz for ^13^C NMR, D_2_O, J in Hz, *δ* in ppm)

Position	R1		X1		A1		C1	
	*δ*_H_	*δ*_C_	*δ*_H_	*δ*_C_	*δ*_H_	*δ*_C_	*δ*_H_	*δ*_C_
1		158.3		158.1		158.0		158.1
2	8.01 (1H, s)	155.6	7.97 (1H, s)	155.3	7.97 (1H, s)	155.3	8.00 (1H, s)	155.3
3		152.0		151.8		151.7		151.7
4		121.4		121.3		121.3		121.3
5	8.31 (1H, s)	142.5	8.32 (1H, s)	142.4	8.04 (1H, s)	142.3	8.31 (1H, s)	142.3
6	5.99 (1H, d, J 6.0)	89.4	5.95 (1H, d, J 6.0)	89.5	6.01 (1H, d, J 8.5)	89.6	5.95 (1H, d, J 6.0)	89.5
7	4.70 (1H, br m)	77.1	4.73 (1H, br m)	77.1	4.80 (1H, br m)	77.1	4.70 (1H, br m)	77.1
8	4.67 (1H, br m)	76.5	4.67 (1H, br m)	76.4	4.73 (1H, br m)	76.3	4.70 (1H, br m)	76.3
9	4.48 (1H, br m)	86.6	4.47 (1H, br m)	86.3	4.55 (1H, br m)	86.4	4.47 (1H, br m)	86.4
10	4.18 (2H, br m)	68.5	4.20 (2H, br m)	68.3	4.28 (2H, br m)	68.4	4.20 (2H, br m)	68.4
11	3.49 (1H, m); 3.77 (1H, m)	74.9	3.50 (1H, m); 3.77 (1H, m)	74.8	3.59 (1H, m); 3.83 (1H, m)	74.7	3.77 (2H, m)	74.8
12		41.2		41.2		41.2		41.2
13	0.83 (3H, s)	23.7	0.82 (3H, s)	23.7	0.91 (3H, s)	23.7	0.82 (3H, s)	23.7
14	0.70 (3H, s)	21.2	0.69 (3H, s)	21.2	0.78 (3H, s)	21.3	0.69 (3H, s)	21.2
15	3.95 (1H, m)	77.1	3.94 (1H, s)	77.0	4.04 (1H, s)	77.1	3.94 (1H, s)	77.1
16		177.6		177.6		177.6		177.6
17	3.39 (2H, m)	38.3	3.38 (2H, m)	38.3	3.48 (2H, m)	38.4	3.39 (2H, m)	38.3
18	2.39 (2H, m)	38.2	2.37 (2H, m)	38.2	2.47 (2H, m)	38.2	2.38 (2H, m)	38.2
19		176.9		176.8		176.8		176.9
20	3.34 (2H, m)	41.5	3.30 (2H, m)	41.6	3.40 (2H, m)	41.7	3.33 (2H, m)	41.6
21	3.05 (2H, m)	31.0	2.99 (2H, m)	30.9	3.08 (2H, m)	30.9	3.02 (2H, m)	30.9
22		196.1		195.9		195.7		195.9
23	6.66 (1H, d, J 16.0)	127.0	6.38 (1H, d, J 15.5)	121.3	6.38 (1H, d, J 16.0)	124.3	6.38 (1H, d, J 16.0)	124.5
24	7.33 (1H, d, J 16.0)	144.5	7.20 (1H, d, J 15.5)	144.5	7.18 (1H, d, J 16.0)	144.7		144.6
25		133.9		128.5		128.9		129.0
26	7.43 (1H, dd, J 1.5, 8.0)	131.3	7.22 (1H, dd, J 1.5, 9.0)	118.7	6.83 (1H, br s)	113.7	6.86 (1H, s)	117.8
27	7.37 (1H, m)	131.9	6.70 (1H, dd, J 1.5, 9.0)	133.5		150.9		150.2
28	7.37 (1H, m)	136.3		161.3		150.2		147.0
29	7.37 (1H, m)	131.9	6.70 (1H, dd, J 1.5, 9.0)	133.5	6.74 (1H, d, 8.5)	118.3	6.72 (1H, d, 8.0)	118.9
30	7.43 (1H, dd, J 1.5, 8.0)	131.3	7.22 (1H, dd, J 1.5, 9.0)	118.7	6.79 (1H, dd, J 1.5, 8.5)	126.5	6.77 (1H, dd, J 1.5, 8.0)	125.9
−OCH3					3.78 (3H, s)	58.5		

The other six Oc4CL proteins, however, showed no reactive action with any substrates. The enzymatic properties were determined by the purified Oc4CL1 with His_6_-tag in N-terminal. The final content of the purified proteins were 0.0808 mg/mL. The optimum pH of the Oc4CL1 was 7.98. It was stable at pH 6–10, and retained more than 85 % activity even at pH 11. The optimal temperature for Oc4CL activity was 30 °C. The enzyme retained 80.80 and 77.44 % even at 40 and 50 °C, respectively. The kinetic parameters of recombinant Oc4CL1 were determined in an enzyme activity assay using compounds **5**–**8** as the substrates, respectively. Kinetic parameters of Oc4CL1 against various phenylpropanoid substrates were listed in Table 3. As showed in Table 3, the best substrate for Oc4CL is *p*-coumaric acid (**6**), with 16.42 μM of *K*_m_ value.

**Table 3:** Enzyme activities of recombinant Oc4CL1

Substrate	*V*_max_ (µM/s)	*V*_max_ (pkat/ug)	*K*_m_ (µM)	k_cat_ (s^−1^)	k_cat_/*K*_m_ (s^−1^ M^−1^)
*Trans*-cinnamic acid	0.175	21.66	108.52	1.29	11,855.05
*p*-coumaric acid	0.356	44.06	16.42	2.62	159,386.65
Caffeic acid	0.546	67.57	79.88	4.01	50,249.26
Ferulic acid	0.917	113.49	87.2	6.74	77,308.62

The *K*_m_ and *V*_max_ of recombinant Oc4CL1 proteins were determined from a Lineweaver–Burk plot

### cDNA isolation and functional characterization of CHS gene family

A CHS gene family harboring three members, *OcCHS1*, *OcCHS2* and *OcCHS3*, was isolated from *O. caudatum* (Table 1). After sequence verification, the three full-length cDNA sequences were inserted into pET-28a (+) to yield recombinant pET-28a (+) derived vectors for heterologous expression, respectively (Additional file 1: Table S2). SDS–PAGE (Additional file 4: Fig. S3) and Western-blot (Additional file 5: Fig. S4) results had indicated the presence of the protein bands representing OcCHS1, OcCHS2 and OcCHS3, respectively. *Trans*-cinnamoyl CoA (**10**), 4-coumaroyl CoA (**11**), caffeoyl CoA (**12**) and feruloyl CoA (**13**) were then added into the crude extracts of three recombinant OcCHSs to attest enzymatic activities. HPLC-DAD results showed there is a new peak in reaction mixture of OcCHS2 when using *trans*-cinnamoyl CoA (**10**) (Additional file 6: Fig. S5) and 4-coumaroyl CoA (**11**) as substrates (Data not shown), respectively. There are no peaks, however, in the reaction mixtures of OcCHS1 and OcCHS3 when the four substrates **10**–**13** were added to the reaction system. LC–MS analyses of these new peaks displayed their [M−H]^−^ ion of *m/z* 255.1 and [M+H]^−^ ion of *m/z* 273.31, corresponding to the calculated mass for pinocembrin chalcone (**4**) and naringenin chalcone (**19**), respectively. 5 mg purified products each were produced by HPLC and applied to NMR. It is hard, however, to get a clear and complete NMR results due to the instability of the two products, pinocembrin chalcone (**4**) and naringenin chalcone (**19**).

**Fig. 4:**
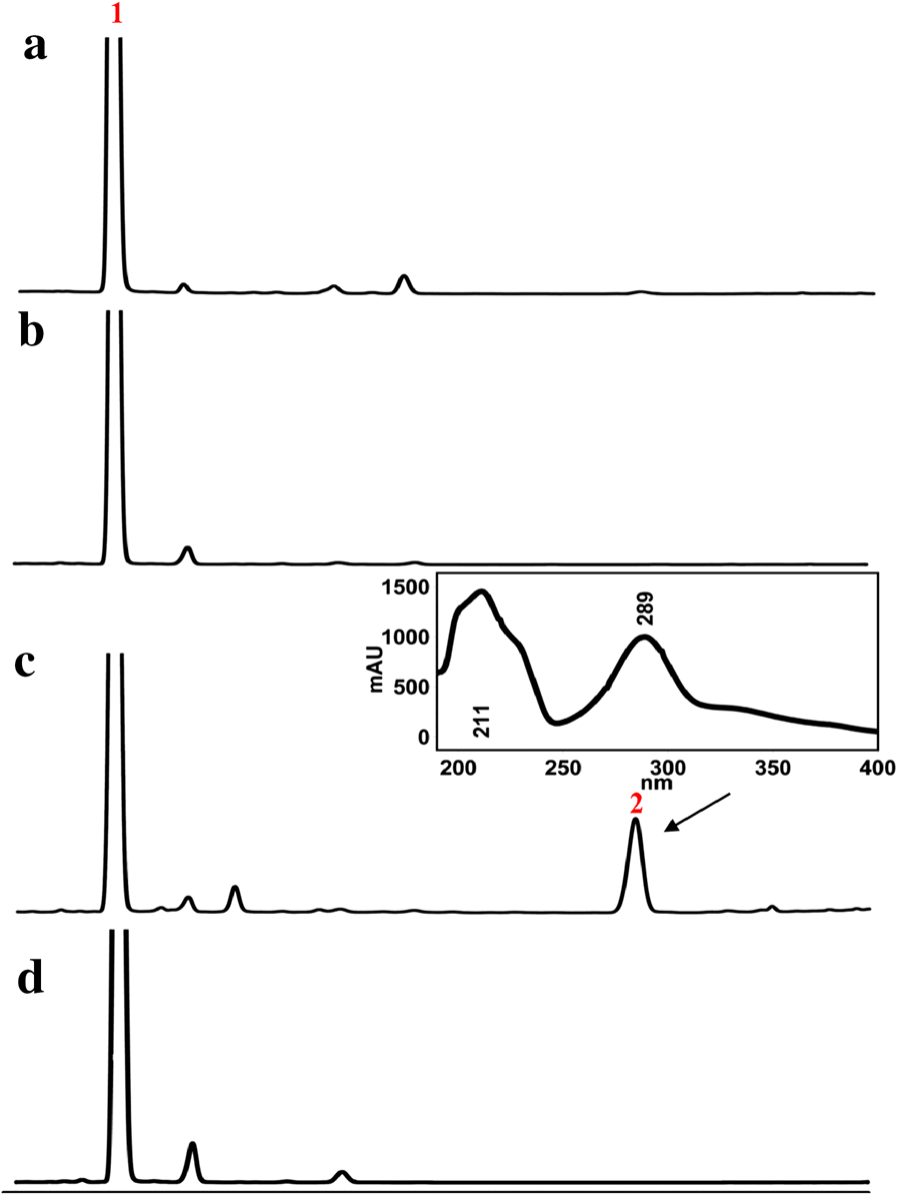
HPLC analyses of fermentation products from engineered strains harboring OcCHSs using *trans*-cinnamic acid (**5**) as the substrate. **a** the blank control of *Trans*etta (DE3); **b** HPLC analysis of fermentation products from strain 1; **c** HPLC analysis of fermentation products from strain 2; **d** HPLC analysis of fermentation products from strain 3; 1 and 2 refer to the substrate *trans*-cinnamic acid (**5**) and the product (*2S*)-pinocembrin (**2**), respectively; the *inserted panel* represented the UV absorbance of the product (*2S*)-pinocembrin (**2**)

**Fig. 5:**
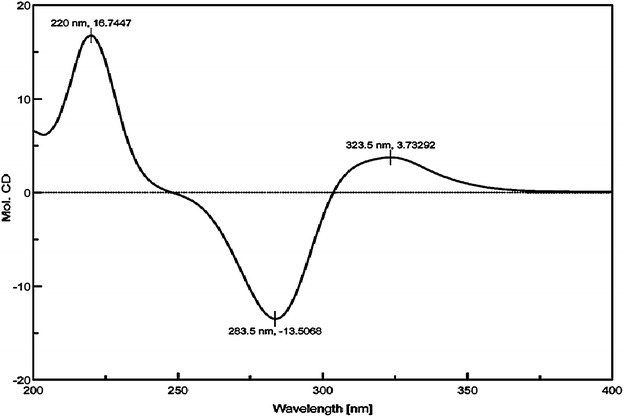
CD spectrum of (*2S*)-pinocembrin (**2**) produced by strain 2

Both of the two chalcones were thought to be rapidly isomerized into corresponding (*2S*)-flavanones [(*2S*)-pinocembrin (**2**) and (*2S*)-naringenin (**21**)], which are stable and can be monitored by HPLC and NMR analysis, under the action of CHI. A new approach based on metabolic engineering, therefore, was applied to functionally characterize *OcCHSs*. Specifically, *OcCHSs* and *MsCHI* (M91079) from *Medicago sativa* L. genes were inserted into pCDFDuet-1 to afford pCDF-OcCHSs-MsCHI (Additional file 1: Table S2). Plasmids pET28a4CL1 and pCDF-OcCHSs-MsCHI were then co-transformed into *E. coli* to form an artificial pathway of (*2S*)-pinocembrin (**2**) biosynthesis. Strain 2 was constructed by grafting the genes coding for *Oc4CL1*, *OcCHS2* and *MsCHI* into *Trans*etta (DE3) (Additional file 1: Table S2). Strain 1 and 3 contained the same set of flavonoid genes as strain2 with the exception of *OcCHS2*, which was respectively replaced by *OcCHS1* and *OcCHS3* (Additional file 1: Table S2). Strains 1–3 were cultured as described previously [19, 20, 37]. When 0.1 mM *trans*-cinnamic acid (**5**) was supplemented in the medium, a new peak with the same retention time and UV spectrum as authentic standard (*2RS*)-pinocembrin (**1**) was reproducibly detected in the engineered strain 2 (Fig. 4). The ion peak [M-H]^−^at *m/z* 255 in the ESI–MS spectra suggested the new compound has a molecular weight of 256, which is consistent with that of authentic (*2RS*)-pinocembrin (**1**). The ^1^H NMR spectrum (Table 4) showed the signals of following protons: *meta*-coupled aromatic protons at δ 5.90 (1H, d, *J* = 2.4 Hz, H-6) and 5.94 (1H, *J* = 2.4 Hz, H-8); a A_2_B_2_X type aromatic protons at δ 7.37 (1H, tr, *J* = 7.8 Hz, H-4′), 7.41 (2H, tr, *J* = 7.8 Hz, H-3′, H-5′), and 7.49 (2H, d, *J* = 7.8 Hz, H-2′, H-6′). Furthermore, three aliphatic doublets at δ 5.46 (1H, dd, *J* = 3.0, 12.0 Hz, H-2), 3.09 (1H, dd, *J* = 12.0, 18.3 Hz, H-3a), and 2.78 (1H, J = 3.0, 18.3 Hz, H-3b), suggesting the presence of a pinocembrin moiety. As shown in Table 4, the ^13^C NMR spectrum presented signals of a carbonyl at δ 196.82 (C-4), and an oxygenated methyne at δ 80.5 (C-2), and a methylene 44.2 (C-3), which were in agreement with the flavanone skeleton. On the basis of the above observations, the structrure of **2** was identified as pinocembrin [14]. The absolute configuration of pinocembrin was further assigned by optical rotation and circular dichroism (CD) spectroscopy. Compared to the control (racemic pinocembrin (**1**) produced by strain 4), the CD spectrum of microbial pinocembrin exhibited a positive cotton effect at 325 nm and a negative cotton effect at 283 nm, which is consistent with the previous report [38]. Therefore, the absolute configuration of the microbial pinocembrin at C-2 was assigned to be *2S*-configured (Fig. 5). This conclusion was further supported by the negative optical rotation ([α]_D_^23^ −22.0°, *c* 1.67 mg/mL, DMSO) of the microbial pinocembrin [14]. Thus, the structure of our microbial pinocembrin was determined to be (*2S*)-pinocembrin (**2**) (Figs. 4, 5; Table 4). No peak, however, was detected in the engineered strains 1 and 3. These results clearly indicated that OcCHS2 was a *bona fide* chalcone synthase.

**Table 4:** ^1^H and ^13^C NMR data for the new fermentation product produced by strain 2 using *trans*-cinnamic acid (**5**) as the substrate (600 MHz for ^1^H NMR and 150 MHz for ^13^C NMR, D_2_O, J in Hz, *δ* in ppm)

Position	Fermentation product of strain 2 using *trans*-cinnamic acid as the substrate		Reference	
	*δ*_H_	*δ*_C_	*δ*_H_	*δ*_C_
2	5.465dd (3.0, 12.0)	80.46	5.46dd (2.4, 12.6)	80.5
3	2.780dd (3.0, 18.3)	44.21	2.78dd (2.4, 16.8)	44.2
	3.094dd (12.6, 18.3)		3.0dd (12.6, 16.8)	
4		197.34		197.3
5		165.51		165.6
6	5.897dd (2.4, 23.1)	97.16	5.90 s	97.3
7		168.42		168.6
8	5.936dd (2.4, 23.1)	96.21	5.94 s	96.3
9		164.68		164.7
10		103.39		103.4
1′		140.45		140.5
2′	7.495d (7.8)	127.35	7.50d (7.8)	127.4
3′	7.415tr (7.8)	129.70	7.42tr (7.8)	129.8
4′	7.368tr (7.8)	129.63	7.36tr (7.8)	129.7

Moreover, strain 2 can also produce a major product, which was characterized as naringenin based on the ESI–MS, UV, and NMR data, when the substrate *p*-coumaric acid (**6**) was added into the culture broth (Additional file 7: Fig. S6; Additional file 1: Table S3).

### cDNA isolation and functional characterization of CHI gene family

**Fig. 6:**
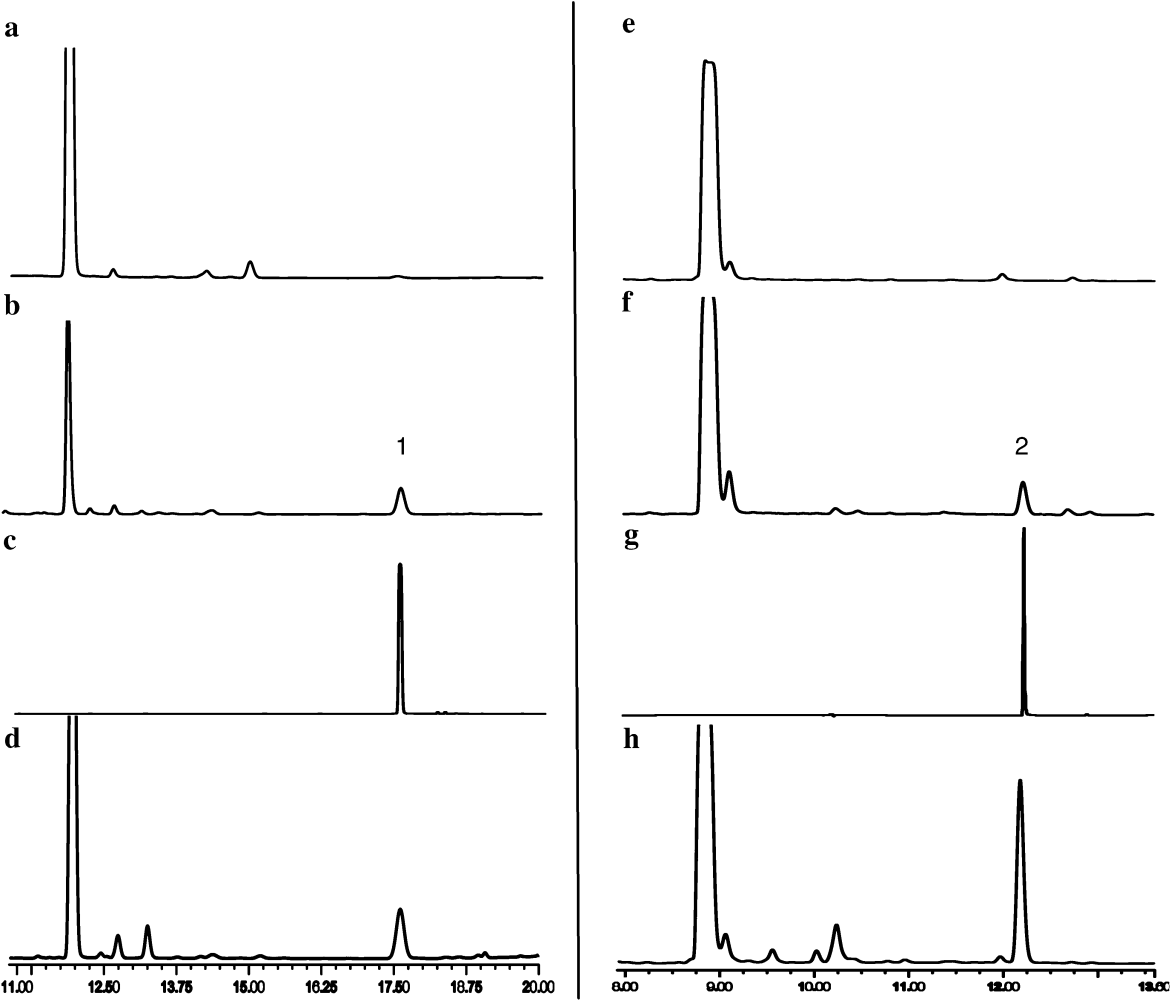
HPLC analysis of the fermentation products from strain 5 using *trans*-cinnamic acid (**5**, *left panel*) or *p*-coumaric acid (**6**, *right panel*) as the the substrate, respectively. ***a&e*** blank control; ***b&f*** HPLC analysis of the fermentation products of strain 5 using *trans*-cinnamic acid (**5**, *left panel*) and *p*-coumaric acid (**6**, *right panel*) as the substrates; ***c&g*** HPLC analysis of the standard pinocembrin and naringenin; ***d&h*** HPLC analysis of the fermentation products of strain 2 using *trans*-cinnamic acid (**5**, *left panel*) and *p*-coumaric acid (**6**, *right panel*) as the substrates; 1 and 2 refer to pinocembrin and naringenin, respectively

A full-length OcCHI cDNA with 633 bp was purified from *O. caudatum* by nested PCR (Table 1). After sequence verification, the resulting PCR fragment was then inserted into pET-28a (+) to acquire the recombinant expression vector pET28aOcCHI after sequence verification. Next, pET28aOcCHI was introduced to *E. coli Trans*etta (DE3) for heterologous expression. SDS–PAGE (Additional file 8: Fig. S7) and western-blot (Additional file 9: Fig. S8) analyses had an indicative result of soluble expression of OcCHI protein. Both pinocembrin chalcone (**4**) and naringenin chalcone (**19**) are the theoretic substrates of OcCHI. Functional identification of OcCHI by in vitro enzymatic reaction was not applicable due to the inaccessibility of the two substrates. A pathway procedure based on metabolic engineering was therefore applied to functionally characterize OcCHI. Specifically, an artificial gene cluster carrying *Oc4CL1*, *OcCHS2* and *OcCHI*, in the form of plasmids pET28a-Oc4CL1 and pCDF-OcCHS2-OcCHI, was transferred to *E. coli* to yield strain 5 (Additional file 1: Table S2). Active OcCHI was reflected by the microbial production of (*2S*)-pinocembrin (**2**). As illustrated in Fig. 6, a new peak was reproducibly appeared in the fermentation products of strain 5 compared to the control. The retention time of the new peak was identical to that of the authentic standard pinocembrin. The compound was then applied to LC–MS analysis in the negative-ion mode. The new compound appeared at *m/z* 255[M-H], indicating that it was pinocembrin. However, the amount of pinocembrin in the supernatant of the cell culture was too small to be preparatively collected for further detection. Moreover, the engineered strain 5 also can produce naringenin after the addition of substrate *p*-coumaric acid (**6**) (Fig. 6)

### Construction and optimization of engineered *E. coli* producing (*2S*)-pinocembrin

A gene cluster containing *Oc4CL*, *OcCHS* and *MsCHI* was introduced into *E. coli* for the purpose of microbial production of (*2S*)-pinocembrin (**2**). Due to higher collaborative efficiency with *Oc4CL1* and *OcCHS2*, *MsCHI* was chosen for further investigation. To test the effect of varied gene organizations in (*2S*)-pinocembrin (**2**) biosynthetic circuit, another engineered strain (strain 6) was also constructed beside strain 2. Strain 6 contained two plasmids, pET28a-Oc4CL1-OcCHS2 and pCDF-MsCHI. Upon IPTG induction, (*2S*)-pinocembrin (**2**) produced by the two engineered *E. coli* cells was analyzed using HPLC. Unexpectedly, only strain 2 can produce 3.58 ± 0.16 mg/L (*2S*)-pinocembrin (**2**) (Table 5). Strains 6 had no flavonoid production.

**Table 5:** Heterologous production of (*2S*)-pinocembrin (**2**) in various engineered strains

Strain	Titre of (*2S*)-pinocembrin (mg/L)			
	No addition of cerulenin	Addition of cerulenin (mM)		
		0.1	0.2	0.3
Strain 2	3.58 ± 0.16			
Strain 7	4.42 ± 0.07			
Strain 8	5.96 ± 0.24	33.20 ± 1.56	36.92 ± 4.10	27.44 ± 3.92
Strain 9	4.13 ± 0.50			
Strain 10	4.77 ± 0.17			
Strain 11	2.77 ± 0.30			

To improve the heterologous expression of pathway enzymes, *Oc4CL1*, *OcCHS2* and *MsCHI* genes were optimized for *E. coli* using the JCat algorithm (http://www.jcat.de/) [39]. These codon-optimized genes were therefore applied to construct five more engineered strains, namely strains 7–11 (Additional file 1: Table S2). These strains were grown in M9 medium with addition of *trans*-cinnamic acid (**5**) and the yield of (*2S*)-pinocembrin (**2**) was compared by HPLC analysis. To test the potential limitations in the engineered pathway, OcCHS2 was first chosen to be highly expressed. As illustrated in Table 5, when condon-optimized *OcCHS2* was introduced into *E. coli*, the resulting strain 7 can produce 4.42 ± 0.07 mg/L (***2S***)-pinocembrin (**2**), 1.23-fold than that of the strain 2 (Table 5). The enhancement of (***2S***)-pinocembrin (**2**) yield in strain 7 was supposed to be the result of overexpression of *OcCHS2*, which leads to a more conversion of pinocembrin chalcone (**4**) from *trans*-cinnamoyl CoA **(10).** To promote the conversion of more (*2S*)-pinocembrin (**2**) from pinocembrin chalcone (**4**), overproduction of CHI is necessary. A condon-optimized *MsCHI* was, therefore, also introduced into the strain 7 to generate the strain 8. As expected, the yield of (***2S***)-pinocembrin (**2**) increased further, reaching to 5.96 ± 0.24 mg/L (Table 5). To direct more *trans*-cinnamoyl CoA **(10)** into (***2S***)-pinocembrin (**2**) biosynthesis, Oc4CL1 was also over-expressed in the strain 10. Unexpectedly, although *Oc4CL1*, *OcCHS2* and *MsCHI* were highly expressed in the strain 10, the yield of (***2S***)-pinocembrin (**2**) in the strain 10 declined to 4.77 ± 0.17 mg/L, only 80 % of that in the strain 8. The decline in production was deemed to result from two kinds of metabolic burden being placed on the cell. One is related to the synthesis of plasmid-encoded proteins. Previous studies indicated that the overproduction of foreign proteins can cause a metabolic load in the host cell, which resulted in a negative effect on *E. coli* cells [40, 41]. In the present investigation, overproduction of three heterologous proteins Oc4CL1, OcCHS2 and MsCHI in strain 10 may impose metabolic burden on the cell, which in turn cause a decline of (***2S***)-pinocembrin (**2**). Moreover, the redundant metabolites in the pathway may lead to the imposition of a metabolic load. In strain 2, the supply of *trans*-cinnamoyl CoA **(10)** was so surplus that can not be completely directed to biosynthesis of (*2S*)-pinocembrin (**2**) by OcCHS2 and MsCHI, even highly expressed OcCHS2 (strain 7) and MsCHI (strain 8). Therefore, the amount of *trans*-cinnamoyl CoA **(10)** accumulated in strain 10 due to overproduction of Oc4CL1, which imposed metabolic burden on *E. coli* cells. This negative effect on *E. coli* strains exerted by metabolites accumulation in turn resulted in the lowered yield of (*2S*)-pinocembrin (**2**). This notion was further supported by the construction of strains 9 and 11. As with that of the strain 10, the amount of *trans*-cinnamoyl CoA **(10)** was kept constant in strains 9 and 11. However, the consumption of *trans*-cinnamoyl CoA **(10)** in both the two strains declined because codon-optimized enzymes were replaced by their normal homologs. Therefore, **c**ompared to the strain 10, more *trans*-cinnamoyl CoA **(10)** was accumulated in strains 9 and 11, which imposed metabolic load on cells and caused a declined (*2S*)-pinocembrin (**2**) production. In the five strains containing codon-optimized pathway enzymes, the strain 8 produced the highest titre of (*2S*)-pinocembrin (**2**). Therefore, the strain 8 was chose to be a start strain for further improvement of (*2S*)-pinocembrin (**2**) production.

Malonyl CoA (**17**) is one of the (*2S*)-pinocembrin (**2**) precursors (Fig. 2). The concentration of malonyl CoA (**17**) in *E. coli* cells was calculated to be only 4–90 mM (0.01–0.23 nmol/mg dry weight) [42]. The low content of intracellular malonyl CoA (**17**) is becoming a bottleneck of (*2S*)-pinocembrin (**2**) yields in engineered *E. coli*. To increase the supply of malonyl CoA (**17**), various concentrations of cerulenin (**18**) (Fig. 2) was supplemented into the fermentative media after the induction period. Cerulenin (**18**) is an antifungal antibiotic produced by *Cephalosporium caerulens*, which blocks fatty acid biosynthesis by inhibiting the β-ketoacyl-acyl carrier protein (ACP) synthases FabB and FabF, thereby preventing channeling of malonyl CoA (**17**) into the pathway for fatty acid synthesis and in turn increasing the supply of malonyl CoA (**17**) to (*2S*)-pinocembrin (**2**) biosynthesis.

As seen in Table 5, addition of cerulenin (**18**) to the strain 8 culture drastically increased its product titers up to 6.2-fold, reaching 36.92 ± 4.1 mg/L (*2S*)-pinocembrin (**2**) at a concentration of 0.2 mM cerulenin (**18**). This result confirmed that the strong metabolic channeling of carbon toward fatty acids is the major competitive step in recombinant (*2S*)-pinocembrin (**2**) biosynthesis. Higher dosing of cerulenin (**18**), however, did not result in more (*2S*)-pinocembrin (**2**) production. When 0.3 mM cerulenin was added into the medium, the titre of (*2S*)-pinocembrin (**2**) decreased to 27.44 ± 3.92 mg/L. This finding suggests accumulation of microbial (*2S*)-pinocembrin (**2**) is not a cerulenin (**18**) dosage-dependent manner. The present investigation, together with previous report [43], indicated that higher supplementation of cerulenin (**18**) resulted in cell death. The detrimental effect may be the main reason of low titre of microbial (*2S*)-pinocembrin (**2**).

## Discussion

Pinocembrin (**1**) is a chiral compound with a chiral center at C-2 (Fig. 1). Chiral pinocembrin (**1**) is a racemic mixture of 2 minor image enantiomers, (*2S*)-pinocembrin (**2**) and (*2R*)-pinocembrin (**3**, Fig. 1). The two enantiomers have shared identical molecular formulas, atom-to-atom linkages, and bonding distances. These identical architectures of these two enantiomers resulted in an often-overlooked chirality analysis of microbial pinocembrin [18–20, 28, 37, 44]. It has long been known that differences in the pharmacokinetic profiles and activity of individual stereoisomers exist, and that these differences can cause significant, sometimes harmful, effects in humans [13, 45]. Thalidomide tragedy is an example [46, 47]. Although it is not sure whether the two enantiomers of pinocembrin have unwanted side effects, it is necessary to analyze the chirality of pinocembrin prior to pharmacological testing. The full characterization of absolute configuration of microbial (*2S*)-pinocembrin (**2**) by combination of MS, NMR, CD and optical detection is thereby uniquely valuable in the present study (Fig. 5; Table 4), which is the first step toward yield improvement and further pharmacological testing.

There are at least three enzymes, such as 4CL, *CHS* and CHI, responsible for (*2S*)-pinocembrin (**2**) biosynthesis from *trans*-cinnamic acid (**5**) (Fig. 2). These three enzymes are encoded by a multi-gene family, respectively. It will take much more time to isolate and further functionally characterize all of these genes by conventional molecular biology technologies. It is particularly important to develop a high-throughput method, allowing for drastically quicker and cheaper genes discovery, and leading towards a far more comprehensive view of biosynthetic pathway of (*2S*)-pinocembrin (**2**) biosynthesis. The advent of next-generation sequencing approach such as transcriptomic analysis provides a platform, which has been proved to be critical in speeding up of the identification of a large number of related genes of secondary products. In the present investigation, a tremendous amount of sequences was yielded by transcriptomic sequencing of *O. caudatum*. A few candidate genes, including *Oc4CLs*, *OcCHSs* and *OcCHIs*, that encode putative enzymes of (*2S*)-pinocembrin (**2**) biosynthetic pathway, were retrived based on the transcriptome analysis (Additional file 1: Table S1). Moreover, in order to quickly construct expression vectors used for heterologous expression of interest genes, an In-Fusion^®^ method based on In-Fusion^®^ enzyme was applied for plasmid construction, which can greatly improve the ligation efficiency of plasmid fragments. These candidate genes were then be functionally identified by combination of in vitro enzymatic reaction and multi-enzyme system based on metabolic engineering in our laboratory. By combination of these biotechnologies, functional characterizations of pathway enzymes of (*2S*)-pinocembrin (**2**) biosynthesis were performed in a rapid fashion, which provides a successful example for gene parts identification used for pathway reconstruction.

In the present investigation, seven full-length 4CL-like cDNA were obtained from *O. caudatum* by nested PCR. The seven genes were thus cloned and the corresponding recombinant proteins (each with an N-terminal His_6_-tag) were expressed in *E. coli* (Additional file 2: Fig. S1, Additional file 3: Fig. S2). In each case, the precise physiological/enzymatic functions of the various 4CL-like members in the *O. caudatum* gene family were carried out using *trans*-cinnamic (**5**), *p*-coumaric (**6**), caffeic (**7**), ferulic (**8**), sinapic (**9**) and benzoic acids (**15**) as potential substrates. The products authenticity in the assay mixtures were verified unambiguously by HPLC analysis rather than by spectrophotometric assays. The data indicated that there was only one *bona fide* 4CL gene, *Oc4CL1*. The result is out of accord with the previous notion that 4CL is encoded by a small multi-gene family [48–51]. The reason why the recombinant Oc4CL2–7 proteins are not active is likely because they can not be actively expressed in *E. coli*. On the other hand, there may be several 4CL genes in *O. caudatum* genome, and we did not isolate all of them and identify enzymatic activity. These 4CL-like proteins were therefore carefully checked for their amino acid sequences (Additional file 10: Fig. S9). Protein sequence alignments of these 4CL-likes revealed the existence of a conserved box I motif (SSGTTGLPKGV), a signature for the superfamily of adenylate-forming enzymes including 4CLs, firefly luciferases, nonribosomal polypeptide synthetases and acyl-CoA synthetases [52, 53]. The absolute conservation of another conserved box II motif (GEICIRG), however, seemed to be restricted to Oc4CL1 and Oc4CL6. The discrepancy of box II in Oc4CL2, 3, 4, 5 and 7 is indicative of their members of the superfamily of adenylate-forming enzymes without 4CL function. Oc4CL6 shared two highly conserved peptide motifs, box I and box II, with Oc4CL1. Oc4CL6, however, differs in four amino acids (Y238F, P278A, M305L and L341I) within a signature motif generally determining 4CL substrate specificity [52], indicative of its devoid of 4CL function (Additional file 10: Fig. S9).

*CHS* is a well-studied ubiquitous plant-specific type III polyketide synthase (PKS) [54–56]. A number of active site residues, including Cys164, *Phe*215, *Phe*265, *His*303 and *Asn*336, are conserved in CHSs but vary in other type III PKSs [54–56]. These conserved amino acids played important roles in *CHS* reaction mechanism. For example, *Phe*265 separates the coumaroyl binding site from the cyclization pocket and may function as a mobile steric gate during successive rounds of polyketide elongation [56]. Single site substitution of these conserved sites is deemed to result in decreased, even no activity. In the present investigation, *Phe*265 of OcCHS2 was replaced by the Ile residue in OcCHS1 and OcCHS3, respectively. The substitution of *Phe*265, therefore, was postulated to be a good explanation of no *CHS* activity of OcCHS1 and OcCHS3 (Additional file 11: Fig. S10).

By combination of in vitro reaction and co-expression assay, we identified the enzymes related to (*2S*)-pinocembrin (**2**) biosynthesis from one single species for the first time. Importantly, as a first step towards microbial scale-up production of (*2S*)-pinocembrin (**2**), combined expressions of these biosynthetic genes in *E. coli* were performed. As illustrated in Table 5, the co-expression of genes originating from single plant species resulted in low-level de novo production of (*2S*)-pinocembrin (**2**). Also, it is clear that the combined use of pathway-encoding genes from the single plant origin does not guarantee the best production of flavonoids [57, 58]. To optimize the (*2S*)-pinocembrin (**2**) production, several parameters have to be considered. First of all, to test the effect of gene organizations on microbial (*2S*)-pinocembrin (**2**) production, two types of gene organizations were generated in two engineered strains. Results indicated only strain 2 can produce (*2S*)-pinocembrin (**2**) (Table 5). No activity of strain 6 is likely to result from inappropriate construction of plasmid pET28a-Oc4CL1-OcCHS2. In this plasmid, Oc4CL1 and OcCHS2 were regulated by their respective expression cassettes. The distance between the two expression cassettes is 14 bp. The short distance was assumed to be the main reason of abnormal transcription or translation of Oc4CL1, or OcCHS2, or both, which was assumed to result in no activity of strain 6. Moreover, the expression levels could be estimated from the gene copy numbers of pathway enzymes. The copy numbers of pCDFDuet-1 (CDF origin) and pET-28a (+) (pBR322 origin) are 20 and 40, respectively. The imbalances within (*2S*)-pinocembrin (**2**) pathway may lead to under-production of pathway enzymes. In addition, we can not rule out the possibility of homologous recombination. Oc4CL1, OcCHS2 and MsCHI were under the control of T7 promoter and RBS (ribosome-binding sequence) in the plasmids pET28a-Oc4CL1-OcCHS2 and pCDF-MsCHI. When the two plasmids were co-transformed into *E. coli*, the resulting strain 6 contained the three repeats of the T7 promoter and RBS. A deletion of the repeats is possible to take place due to possibly homologous recombination. The productivity is still low although strain 2 was detected to produce (*2S*)-pinocembrin (**2**). We hypothesized that the low titer of (*2S*)-pinocembrin (**2**) production from recombinant *E. coli* is partially due to the low activity of pathway enzymes. Oc4CL1, OcCHS2 and MsCHI, therefore, were designed to optimize the codon usage for *E. coli*. The enhancements of (*2S*)-pinocembrin (**2**) titre was observed in all the strains containing *E. coli*-preferred genes with the exception of strain 11. Unexpectedly, when co-expression of synthetic condon-optimized *Oc4CL1*, *OcCHS2* and *MsCHI* was performed in strain 11, decreased yield in (*2S*)-pinocembrin (**2**) was observed (Table 5). Typically, codon-optimization of *Oc4CL1*, *OcCHS2* and *MsCHI* may lead to their over-expression in the strain 11. Overproduction of the three heterologous proteins, however, usually imposes the metabolic burden on the strain and in turn results in the negative effect on cell physiology. Hence, it is supposed that lowered yield of (*2S*)-pinocembrin (**2**) in strain 11 should be caused by overproduction of heterologous *Oc4CL1*, *OcCHS2* and *MsCHI.* Overall, an engineered strain, strain 8 with higher titre of 5.96 ± 0.24 mg/L (*2S*)-pinocembrin (**2**) was selected for further improvement. At this stage, insufficient levels of the precursor malonyl CoA (**17**) could be limiting for the overall product titers. In order to find out whether the availability of malonyl CoA (**17**) was limiting, cultivations were performed in which cerulenin (**18**, up to 0.3 mM) was supplemented during the production phases. The exclusive supplementation of 0.2 mM cerulenin (**18**) drastically increased product titers up to 6.2-fold, reaching 36.92 ± 4.1 mg/L, which was comparable to that of the previous reports (Table 5) [18, 19].

Although the yields of (*2S*)-pinocembrin (**2**) in *E. coli* were increased, there is still room for improvement. Common methods used to improve production from engineered biosynthetic pathways include, but is not limited to, enhancing production of pathway enzymes [19, 20, 37], yield enhancement of the intracellular pool of precursors [19, 59] and balancing multi-gene expression to optimize flux [18, 57, 60]. It is well recognized that optimal protein yield may be achieved either by mutagenic experiments to create the desired attributes of an enzyme or through selection of variant enzymes deposited in public databases with differing kinetic properties. Typically, codon optimization had been proved to be a mutagenesis technique improving the efficiency of heterologous protein production in the present and previous studies [18, 57, 60]. Also, screening various target enzymes with desired attributes from the public databases can optimize engineered pathways. There are many well-characterized homologs of 4CL [48, 61, 62], *CHS* [63, 64] and CHI [65] in publicly available sequence databases. These variants have differing kinetic properties. They may be chosen to investigate their in vivo performance for (*2S*)-pinocembrin (**2**) production in the context of the entire (*2S*)-pinocembrin (**2**) pathway. The best performing variants will be used as the ideal candidates for (*2S*)-pinocembrin (**2**) production.

Addition of cerulenin (**18**) can improve the productivity of (*2S*)-pinocembrin (**2**), however, the high cost of cerulenin (**18**) prohibits its use in industrial-scale fermentations. Other additional strategies, like reconstruction of malonate assimilation pathway containing two components of matB (encoding malonyl-CoA synthetase) and matC (encoding malonate carrier protein) [18, 66], overexpression of multisubunit complex of acetyl-CoA carboxylase (ACC) [19, 66, 67] and genetic modification in acetate assimilation pathways [66, 67], were pursued for improving the intracellular malonyl CoA (**17**) availability in *E. coli* to circumvent cerulenin (**18**) addition.

In the expression of a multi-gene heterologous pathway, the activity of a single enzyme may be out of balance with that of the other enzymes in the pathway, leading to unbalanced carbon flux and the accumulation of an intermediate. Varied strategies, like modular metabolic strategy [18, 60] and expression correlation analysis [57], may be employed to balance the overall pathway.

Besides, selection of appropriate hosts [60], alleviation of the metabolic burden [60] and optimization of fermentation conditions [60] should be taken into account since they may lead to robust improvement of (*2S*)-pinocembrin (**2**) produced. Availability of such a powerful *E. coli* platform paves the way for scale-up production and eventual industrialization of (*2S*)-pinocembrin (**2**) production.

## Conclusions

In the present study, we presented a full characterization of absolute configuration of microbial (*2S*)-pinocembrin (**2**), a chiral molecule with versatile pharmacological and biological activities. Also, we isolated and functionally identified gene parts used for pathway reconstruction of (*2S*)-pinocembrin (**2**) biosynthesis in *E. coli* based on transcriptome-wide sequencing in this investigation. The resulting engineered *E. coli* can produce 36.92 ± 4.1 mg/L (*2S*)-pinocembrin (**2**), which paves the way for yield increase and further pharmacological testing of chiral (*2S*)-pinocembrin (**2**).

## Methods

### Chemicals and enzymes

*Trans*-cinnamic acid (**5**), *p*-coumaric acid (**6**), caffeic acid (**7**), ferulic acid (**8**), sinapic acid (**9**) and benzoic acid (**15**) were obtained from Sigma-Aldrich Co.LLC (St. Louis, MO, United States). Racemic pinocembrin (**1**) was kindly presented by Prof. Zhang TT of IMM (Institute of Materia Medica), China. Cerulenin (**18**), used for malonyl CoA (**17**) availability experiments, was purchased from J&K Scientific Ltd (Beijing, China). In-Fusion^®^ HD Cloning Kit and restriction enzymes were purchased from Takara Shuzo Co. Ltd (Kyoto, Japan). KOD Plus Taq DNA polymerase was purchased from Toyobo Co. Ltd (Osaka, Japan). All other fine chemicals are analytical grade.

### Strains and plasmids

pEASY™-T1 Simple vector was from TransGen Co. Ltd (Beijing, China).The *E. coli* strain Trans1-T1 and *Trans*etta(DE3) (TransGen Co. Ltd) were used as a bacterial host for recombinant plasmid amplification and enzyme expression, respectively. The strain was grown in Luria–Bertani medium (10 g/L Bacto-Tryptone, 5 g/L Bacto-yeast extract, 10 g/L NaCl) supplemented with ampicillin (100 μg/mL) when required for selection.

The expression vector pET-28a (+) and pCDFDuet-1 were from Novagen (Madison, USA) and used for heterologous expression. The plasmids and strains used in this study are provided in Additional file 1: Table S2.

### Plant materials

*O. caudatum* plants were grown under sterile conditions on 67-V medium [68] at a temperature of 22 °C and 16 h light/8 h dark cycle. The bulbs of *O. caudatum* were collected and used fresh or were frozen in liquid N_2_ and stored at −80 °C for RNA isolation.

### Transcriptome sequencing and analysis

The detailed procedure is the same as the previous reports by our laboratory [69–71]. Specifically, a (cDNA) sequencing library was prepared from the total RNA of *O. caudatum* using a mRNA-seq Sample Preparation Kit (Illumina) following the manufacturer’s protocol. After that, the resultant cDNA library could be sequenced using Illumina HiSeq™ 2000. Short nucleotide reads obtained via Illumina sequencing were assembled by the Trinity software to produce error-free, unique contiguous sequences (contigs). Then, these contigs were connected to acquire non-redundant unigenes, which could not be extended on either end.

After transcriptome sequencing of *O. caudatum*, the resulting unigenes were aligned by BLAST X to protein databases like nr, Swiss-Prot, KEGG and COG (e < 0.00001), and aligned by BLAST N to nucleotide databases nt (e < 0.00001), retrieving proteins with the highest sequence similarity with the given unigenes along with their protein functional annotations. The candidate unigenes which were assigned to (*2S*)-pinocembrin (**2**) biosynthesis pathway based on KEGG pathway analysis, that is 4CL-like (Oc4CLs), *CHS*-like (OcCHSs) and CHI-like homologs (OcCHIs), were retrieved for further studies.

### cDNA isolation and functional characterization of 4CL gene family

Since the assembled sequences were products of de novo assemblies, they were considered prone to error. To confirm that the sequences represented true gene products, experimental verifications were performed by designing gene-specific primers for these full-length sequences encoding (*2S*)-pinocembrin (**2**) pathway enzymes and verifying the identity of amplified products by sequencing of the PCR amplimers. All the oligonucleotides used for DNA manipulation are described in the Additional file 1: Table S4.

Amplification of full-length cDNA synthesized from mRNA extracted from the sterile bulb tissues of *O. caudatum* was performed by a nested PCR method. The amplified products were inserted in pEASY™-T1 Simple vector for sequencing.

After sequence verifications, these full-length cDNAs were inserted into *Eco*RI/*Hin*dIII linearized pET-28a (+) using In-Fusion^®^ technology for heterologous expression as the procedures previously described [69–71]. In all cases, successful gene cloning was verified by digestion checks, and the absence of undesired mutations introduced during PCR was verified by direct nucleotide sequencing.

Induction of Oc4CL proteins expression was carried out at 27 °C for 8 h after addition of IPTG with a final concentration of 0.4 mM. His-tag recombinant Oc4CL proteins were subsequently purified using immobilized metal affinity chromatography system. Activity assay and biochemical properties analysis of the recombinant proteins were performed differentially. 4CL activity was determined by measuring the formation of the corresponding CoA thioesters from *trans*-cinnamic acid (**5**) and its derivatives by in vitro reaction. 100 µL crude protein extracts for Oc4CLs (derived from 1 mL of culture) was added to the reaction mixture containing 2.5 mM MgCl_2_, 2.5 mM ATP and 20 µM substrates (*trans*-cinnamic acid (**5**), *p*-coumaric acid (**6**), caffeic acid (**7**), ferulic acid (**8**), sinapic acid (**9**) and benzoic acid (**15**), respectively) in 200 mM Tris-HCl (pH 7.9) in a total volume of 1000 μL. The reaction was started by the addition of 0.2 mM CoA. The crude protein extracts prepared from *E. coli* [pET28a] and heat denatured protein extracts derived from *E. coli* [pET28a-Oc4CLs] were used as the controls. After incubated at 30 °C for 15 min, 40 μL of acetic acid were added to terminate the reaction. The formation of CoA esters was unambiguously determined by HPLC-UV, HPLC-MS and ^1^H and ^13^C NMR. HPLC was performed on a HITACHI instrument using a C18 column [YMC-Pack ODS-A (5 µm, 12 nm) 250 × 4.6 mm I.D]. Chromatographic condition was as follow. Mobile phase (A): 50 mM NH_4_Ac water (pH 4.6); (B): 100 % acetonitrile; gradient elute for 0–7 min: B 2–50 %, 7–15 min: B 50–50 %, 15~18 min: B 50–100 %; flow rate: 1 mL/min; column temperature: 25 °C; sample size: 25 μL. The detection was made on a UV detector at 320 nm for enzymatic products of *p*-coumaric acid (**6**), caffeic acid (**7**), ferulic acid (**8**) and sinapic acid (**9**), 270 nm for reaction product of *trans*-cinnamic acid (**5**) and 259 nm for benzoic acid (**15**).

LC–MS analysis was performed using an Agilent 1200 RRLC series HPLC system (Agilent Technologies, Waldbronn, Germany) coupled to the QTRAP MS spectrometer (QTRAP 2000, Applied Biosystems/MDS SCIEX) tandem mass spectrometer equipped with a Turbo Ion spray ion source (Concord, ON, Canada) which was controlled by Analyst 1.5. UV spectra were recorded from 190 to 400 nm. The mass spectrometer was operated in negative ion mode and spectra were collected in the enhanced full mass scan mode from *m/z* 100 to 1000.

NMR spectroscopic data were obtained at 500 MHz for ^1^H NMR and 125 MHz for ^13^C NMR using the solvent CDCl_3_ on Bruker-500 spectrometers, respectively. Chemical shifts (δ) are given in ppm, coupling constants (J) are given in hertz (Hz).

To examine the biochemical properties and kinetic parameters of Oc4CL1, purified recombinant protein was used. The pH optimum was determined in a buffer of 200 mM Tris-HCl containing 20 μM varied substrates, 2.5 mM ATP, 25 mM MgCl_2_, and 0.02 mM CoA, in the pH range from 5.90 to 9.48 using 1.616 μg pure enzyme in a final volume of 200 μL. Samples were incubated at 30 °C for 2 min.

To determine the optimum temperature, assays were performed in the buffer of 200 mM Tris-HCl containing 20 μM diverse substrates, 2.5 mM ATP, 25 mM MgCl_2_, and 0.02 mM CoA at pH 7.9 for 2 min with various temperatures from 15~50 °C.

Kinetic analysis of Oc4CL1 was conducted by the standard assay with a range of concentrations of different substrates. The apparent K_m_ (Michaelis–Menten constant) and the maximum rate of OC4CL1 (V_max_) were determined graphically by the Lineweaver–Burk plot.

### cDNA isolation and functional characterization of *CHS* gene family

The full-length cDNAs of candidate *CHS* genes were isolated from *O. caudatum* by nested PCR using the gene-specific primers (Additional file 1: Table S4). The resulting PCR products were cloned into pEASY™-T1 Simple vector to generate pEASYOcCHSs and verified by sequencing (Additional file 1: Table S2). After confirming the sequences fidelity, the three OcCHS genes were functionally identified either by in vitro reaction or by multienzyme-cooperative systems. In vitro enzymatic reaction is a simple and direct way to identify gene function. Specifically, three OcCHS genes were subcloned in frame with the polyhistidine tag into the *Bam*HI/*Hin*dIII sites of pET-28a (+), giving three constructs, pET28aOcCHS1~3. Heterologous expression, SDS–PAGE analysis and western-blot verification of the recombinant OcCHS proteins were performed using the same procedures as that of Oc4CLs. After induction by the addition of IPTG, 1 ml cells were harvested by centrifugation at 10,000*g* for 2 min at 4 °C. The resulting cell pellets were resuspended in 1 ml of 200 mM Tris-HCl (pH 7.9) and disrupted by sonication. Cell debris was removed by centrifugation at 12,000*g* for 5 min at 4 °C, and the resulting supernatant was used as crude protein extracts for in vitro activities of the recombinant OcCHS proteins. OcCHS activities were determined by measuring the formation of the corresponding chalcones from CoA thioesters. Enzyme activities were carried out at 30 °C for 30 min in 1 ml of 200 mM Tris-HCl (pH 7.9) containing 0.2 mM CoA thioesters and 20 μM malonyl-CoA. The reactions were terminated by adding 40 μl acetic acid and then extracted three times with 1.5 ml ethyl acetate. After vortexing and centrifugation (12,000*g*, 10 min), the top organic layer was separated and evaporate to dryness, and then the remaining residue was resolubilized with 250 μl methanol. The resulting methanol samples were then analyzed by HPLC and LC–MS using the same program as that for Oc4CLs. UV detection was performed at 341 nm. The function of OcCHSs was also characterized using multienzyme-cooperative systems owing to the unstability of pinocembrin chalcone (**4**), a product to *CHS* reaction. Specifically, The candidate OcCHSs were co-expressed with a Oc4CL1 and chalcone isomerase from *Medicago sativa* (MSCHI, GenBank accession number M91079) [27, 31, 72, 73] in *E. coliTrans*etta (DE3) to form a (*2S*)-pinocembrin (**2**) biosynthetic pathway. The instable pincembrin chalcone (**4**) produced by *CHS* was then biotransformed into (*2S*)-pinocembrin (**2**), which was validated by HPLC analysis, under the action of MsCHI.

First of all, a synthetic *MsCHI* was inserted into *Bam*HI/*Hin*dIII sites of pCDFDuet-1, resulting in pCDF-MsCHI. *OcCHSs* genes were PCR amplified from respective pET28a-derived plasmids and were then ligated into pCDF-MsCHI between *Nde*I and *Xho*I sites, yielding pCDF-OcCHSs-MsCHI (OcCHSs refer to OcCHS1, OcCHS2 and OcCHS3). Both *OcCHS* and *MsCHI* were separately placed under the control of the T7 promoter of pCDFDuet-1.

Corresponding plasmids were transformed into *E. coliTrans*etta (DE3). The resulting three *E. coli* recombinant strains, strains 1–3, harboring either of the plasmids pCDF-OcCHS1-MsCHI, pCDF-OcCHS2-MsCHI or pCDF-OcCHS3-MsCHI together with plasmid pET28a-Oc4CL1 were used for shake flask experiments in 50 mL M9 minimal medium with addition of 0.1 mM *trans*-cinnamic acid (**5**) as previous described (Additional file 1: Table S2) [31]. Incubation continued at 30 °C for 36 h prior to analysis of fermentation products. To analyze flavonoid production, *E. coli* cells were separated through centrifugation (8000 rpm, 10 min, 4 °C). After extraction of the supernatant with an equal volume of ethyl acetate three times, the resulting top organic layer were concentrated by evaporation and dissolved in 200 μL of methanol. 20 μL of this was injected into HPLC for UV spectra and mass spectrometer analysis using the same procedure mentioned above with the exception of monitoring absorbance at 290 nm. Absolute configuration of the fermentation product was further fully characterized by combination of ^1^H and ^13^C NMR (600 MHz), CD spectroscopy and optical detection. Also, the strain 4, harboring plasmids pET28a-Oc4CL1 and pCDF-OcCHS2, was constructed for microbial production of racemic pinocembrin (**1**) used as the control for absolute configuration characterization of (*2S*)-pinocembrin (**2**).

### cDNA isolation and functional characterization of CHI gene family

*OcCHI* gene isolation and protein expression were performed using the same procedure as described above. The functional characterization of *OcCHI* was also performed using multienzyme-cooperative systems due to the unavailable pinocembrin chalcone (**4**), a substrate of OcCHI. An artificial gene cluster containing *Oc4CL1*, *OcCHS2* and *OcCHI* was grafted to *E. coliTrans*etta (DE3) to rebuild a (*2S*)-pinocembrin (**2**) pathway. The OcCHI activity is reflected by the production of (*2S*)-pinocembrin (**2**). To this end, a plasmid named pCDF-OcCHS2-OcCHI was constructed by replacement of *MsCHI* with *OcCHI* in the parental vector pCDF-OcCHS2-MsCHI (Additional file 1: Table S2). Plasmids pCDF-OcCHS2-OcCHI and pET28aOc4CL1 were then co-transformed into *Trans*etta (DE3) to achieve strain 5 (Additional file 1: Table S2). The fermentation procedure, as well as the HPLC analysis and structural characterization of the fermentation products was same as described above.

### Construction and optimization of engineered *E. coli* producing (*2S*)-pinocembrin

To test the effect of coordinated expression of pathway enzymes on (*2S*)-pinocembrin (**2**) production, another plasmid, pET28a-Oc4CL1-OcCHS2, was constructed (Additional file 1: Table S2). The detailed procedure is as follows. *OcCHS*2 expression cassette containing T7 promoter, RBS and *OcCHS*2 ORF was PCR amplified from pET28a-OcCHS2. The resulting PCR fragment was inserted into pET28a-Oc4CL1 between restriction sites *Bgl*II/*Sph*I generating pET28a-Oc4CL1-OcCHS2. Plasmids pET28a-Oc4CL1-OcCHS2 and pCDF-MsCHI were then co-introduced into *Trans*etta (DE3) to create strain 6 (Additional file 1: Table S2). In strain 2, *Oc4CL1* was placed in different plasmids from *OcCHS2* and *MsCHI*. *Oc4CL1* and *OcCHS2*, however, were placed in the pET-28a (+), which is different from *MsCHI*, in strain 6.

In an attempt to increase the expression level of (*2S*)-pinocembrin (**2**) pathway enzymes, *Oc4CL1*, *OcCHS2* and *MsCHI* were codon-optimized for *E. coli* expression (http://www.jcat.de/), respectively. In future references, synthetic genes/proteins are denoted by a prefix ‘‘opt’’. Three more plasmids pET28a-OptOc4CL1, pCDF-OptOcCHS2-MsCHI and pCDF-OptOcCHS2-OptMsCHI, carrying synthetic codon optimized *Oc4CL1* (*OptOc4CL1*), *OcCHS2* (*OptOcCHS2*) and *MsCHI* (*OptMsCHI*), were in turn generated using the same procedure that was used to generate their parental plasmids pET28a-Oc4CL1 and pCDF-OcCHS2-MsCHI (Additional file 1: Table S2). Varied plasmids combinations were introduced into *Trans*etta (DE3) to generate strains 7–11 (Additional file 1: Table S2).

Moreover, to improve malonyl CoA (**17**) availability, varied concentrations of cerulenin (**18**) (0.1, 0.2 and 0.3 mM) were added into medium to culture strain 8 (Additional file 1: Table S2) [74–76]. The culture of these strains, extraction, HPLC analysis, and fully structural characterizations of fermentation products were performed as described above. The flavonoid productions from the various recombinant strains were presented as the averages of three independent experiments.

## Additional files

10.1186/s12934-016-0424-8 Unigenes assigned to every step of (*2S*)-pinocembrin(**2**) biosynthetic pathway. **Table S2.** Plasmids and strains used in this study. **Table S3.** ^1^H and ^13^C NMR data for the new fermentation product produced by strain 2 using *p*-coumaric acid (**6**) as the substrate (600 MHz for ^1^H NMR and 150 MHz for ^13^C NMR, D_2_O, J in Hz, *δ* in ppm). **Table S4.** Oligonucleotides used in this investigation.
10.1186/s12934-016-0424-8 SDS-PAGE of crude protein extracts from a transformant expressing Oc4CL1 (Panel A, lane 1), Oc4CL2 (Panel B, lane 1), Oc4CL3 (Panel C, lane 1), Oc4CL4 (Panel D, lane 1), Oc4CL5 (Panel E, lane 1), Oc4CL6 (Panel F, lane 1), Oc4CL7 (Panel G, lane 1), an empty vector transformant (pET-28a (+), lane 2). M stands for molecular weight markers. The migration positions of standards (in kDa) are shown at left and the red arrows indicate the recombinant Oc4CL proteins.
10.1186/s12934-016-0424-8 Western blot analysis of crude protein extracts from a transformant expressing Oc4CL1 (Panel A, lane 1), Oc4CL2 (Panel B, lane 2), Oc4CL3 (Panel C, lane 3), Oc4CL4 (Panel D, lane 4), Oc4CL5 (Panel E, lane 5), Oc4CL6 (Panel F, lane 6), Oc4CL7 (Panel G, lane 7), an empty vector transformant (pET-28a (+), lane CK).
10.1186/s12934-016-0424-8 SDS-PAGE of total protein isolated from *E. coli* expressing OcCHS1 (panel A, lane 1), OcCHS2 (panel B, lane 2), OcCHS3 (panel C, lane 3) or control empty vector (Lane CK). M stands for molecular weight markers. The migration positions of standards (in kDa) are shown at left and the red arrows indicate the recombinant OcCHS proteins.
10.1186/s12934-016-0424-8 Western blot analysis of total protein isolated from *E. coli* expressing OcCHS1 (lane 1), OcCHS2 (lane 2), OcCHS3 (lane 3) or control empty vector (Lane CK).
10.1186/s12934-016-0424-8 HPLC analysis of the reaction products of recombinant OcCHS2 protein. A, HPLC analysis of the reaction product from *E.coli*[pET28a]; B, HPLC analysis of the reaction products of recombinant Oc4CL1 protein using *trans*-cinnamic acid (**5**) as the substrate; C, HPLC analysis of the reaction products of recombinant OcCHS2 protein using reaction products of recombinant Oc4CL1 as the substrate. peak 1, *trans*-cinnamic acid (**5**); peak 2, *trans*-cinnamoyl CoA (**10**), peak 3, pinocembrin chalcone (**4**).
10.1186/s12934-016-0424-8 HPLC analysis of the fermentation products of strains 1-3. A, HPLC analysis of the fermentation products of strain *E. coli*[pET28a] using *p*-coumaric acid (**6**) as the substrate; B, HPLC analysis of the fermentation products of strains 1 using *p*-coumaric acid (**6**) as the substrate; C, HPLC analysis of the fermentation products of strains 2 using *p*-coumaric acid (**6**) as the substrate; D, HPLC analysis of the fermentation products of strains 3 using *p*-coumaric acid (**6**) as the substrate; peak 1, *p*-coumaric acid (**6**); peak 2, naringenin; The inserted tablet represented the UV absorbance of the product naringenin.
10.1186/s12934-016-0424-8 SDS-PAGE analysis of total proteins stained with silver nitrate. Lane 1, total protein from *E. coli* expressing OcCHI; lane CK, total protein from bacteria containing the empty vector alone; Molecular masses of markers are shown to the left in kDa (lane M). The red arrows indicate the recombinant OcCHI protein.
10.1186/s12934-016-0424-8 Western blot analysis of total protein isolated from *E. coli* expressing OcCHI (lane 1) and control empty vector (Lane CK).
10.1186/s12934-016-0424-8 Sequences alignment of *Oc4CLs*. The conserved putative AMP-binding motif (Box I) and the putative catalytic motif GEICIRG (Box II) is highlighted by red square. 12 amino acids proposed to function as a 4CL substrate specificity code are labelled with solid circles. The mutated amino acids between Oc4CL1 and Oc4CL6 are red shaded.
10.1186/s12934-016-0424-8 Sequences alignment of *OcCHS* proteins. Five conserved amino acids are labelled with solid circles. The mutated amino acid in OcCHS1 and OcCHS3 are red shaded.

## Abbreviations

accABCD: acetyl-CoA carboxylase complex
C4H: *trans*-cinnamic acid 4-hydroxylase
4CL: 4-coumarate:coenzyme A ligase
CHS: chalcone synthase
CHI: chalcone isomerase
FabA: 3-hydroxydecanoyl-ACP dehydrase
FabB: β-ketoacyl-ACP synthase I
FabD: malonyl-CoA:ACP transacylase
FabF: β-ketoacyl-ACP synthase II
FabG: β-ketoacyl-ACP reductase
FabH: β-ketoacyl-ACP synthase III
FabI: enoyl-ACP reductase
FabZ: β-hydroxyacyl-ACP dehydratase
matB: malonyl-CoA synthetase
matC: malonate carrier protein
SDS–PAGE: sodium dodecyl sulfate polyacrylamide gel electrophoresis
IPTG: isopropyl-β-d-thiogalactopyranoside
HPLC-DAD: high-performance liquid chromatography-diode array detector
