# Supplementary material for: Transcriptome-enabled discovery and functional characterization of enzymes related to (2S)-pinocembrin biosynthesis from Ornithogalum caudatum and their application for metabolic engineering
Source: Microb Cell Fact. 2016 Feb 4;15:27. doi: 10.1186/s12934-016-0424-8 (PMC4743118; doi:10.1186/s12934-016-0424-8)
Supplement: Supplementary file 1 — 10.1186/s12934-016-0424-8 Unigenes assigned to every step of (2S)-pinocembrin(2) biosynthetic pathway. Table S2. Plasmids and strains used in this study. Table S3. 1H and 13C NMR data for the new fermentation product produced by strain 2 using p-coumaric acid (6) as the substrate (600 MHz for 1H NMR and 150 MHz for 13C NMR, D2O, J in Hz, δ in ppm). Table S4. Oligonucleotides used in this investigation. [file 12934_2016_424_MOESM11_ESM.doc]

Fig.S10
