# Supplementary material for: Transcriptome-enabled discovery and functional characterization of enzymes related to (2S)-pinocembrin biosynthesis from Ornithogalum caudatum and their application for metabolic engineering
Source: Microb Cell Fact. 2016 Feb 4;15:27. doi: 10.1186/s12934-016-0424-8 (PMC4743118; doi:10.1186/s12934-016-0424-8)
Supplement: Supplementary file 2 — 10.1186/s12934-016-0424-8 SDS-PAGE of crude protein extracts from a transformant expressing Oc4CL1 (Panel A, lane 1), Oc4CL2 (Panel B, lane 1), Oc4CL3 (Panel C, lane 1), Oc4CL4 (Panel D, lane 1), Oc4CL5 (Panel E, lane 1), Oc4CL6 (Panel F, lane 1), Oc4CL7 (Panel G, lane 1), an empty vector transformant (pET-28a (+), lane 2). M stands for molecular weight markers. The migration positions of standards (in kDa) are shown at left and the red arrows indicate the recombinant Oc4CL proteins. [file 12934_2016_424_MOESM1_ESM.doc]

Supplementary figure legends

**Fig. S1** SDS-PAGE of crude protein extracts from a transformant expressing Oc4CL1 (Panel A, lane 1), Oc4CL2 (Panel B, lane 1), Oc4CL3 (Panel C, lane 1), Oc4CL4 (Panel D, lane 1), Oc4CL5 (Panel E, lane 1), Oc4CL6 (Panel F, lane 1), Oc4CL7 (Panel G, lane 1), an empty vector transformant (pET-28a (+), lane 2).

M stands for molecular weight markers. The migration positions of standards (in kDa) are shown at left and the red arrows indicate the recombinant Oc4CL proteins.

**Fig. S2** Western blot analysis of crude protein extracts from a transformant expressing Oc4CL1 (Panel A, lane 1), Oc4CL2 (Panel B, lane 2), Oc4CL3 (Panel C, lane 3), Oc4CL4 (Panel D, lane 4), Oc4CL5 (Panel E, lane 5), Oc4CL6 (Panel F, lane 6), Oc4CL7 (Panel G, lane 7), an empty vector transformant (pET-28a (+), lane CK).

**Fig. S3** SDS-PAGE of total protein isolated from *E. coli* expressing OcCHS1 (panel A, lane 1), OcCHS2 (panel B, lane 2), OcCHS3 (panel C, lane 3) or control empty vector (Lane CK).

M stands for molecular weight markers. The migration positions of standards (in kDa) are shown at left and the red arrows indicate the recombinant OcCHS proteins.

**Fig. S4** Western blot analysis of total protein isolated from *E. coli* expressing OcCHS1 (lane 1), OcCHS2 (lane 2), OcCHS3 (lane 3) or control empty vector (Lane CK).

**Fig.S5** HPLC analysis of the reaction products of recombinant OcCHS2 protein.

A, HPLC analysis of the reaction product from *E.coli*[pET28a]; B, HPLC analysis of the reaction products of recombinant Oc4CL1 protein using *trans*-cinnamic acid (**5**) as the substrate; C, HPLC analysis of the reaction products of recombinant OcCHS2 protein using reaction products of recombinant Oc4CL1 as the substrate. peak 1, *trans*-cinnamic acid (**5**); peak 2, *trans*-cinnamoyl CoA (**10**), peak 3, pinocembrin chalcone (**4**)

**Fig.S6** HPLC analysis of the fermentation products of strains 1-3.

A, HPLC analysis of the fermentation products of strain *E. coli*[pET28a] using *p*-coumaric acid (**6**) as the substrate; B, HPLC analysis of the fermentation products of strains 1 using *p*-coumaric acid (**6**) as the substrate; C, HPLC analysis of the fermentation products of strains 2 using *p*-coumaric acid (**6**) as the substrate; D, HPLC analysis of the fermentation products of strains 3 using *p*-coumaric acid (**6**) as the substrate; peak 1, *p*-coumaric acid (**6**); peak 2, naringenin; The inserted tablet represented the UV absorbance of the product naringenin.

**Fig. S7** SDS-PAGE analysis of total proteins stained with silver nitrate. Lane 1, total protein from *E. coli* expressing OcCHI; lane CK, total protein from bacteria containing the empty vector alone; Molecular masses of markers are shown to the left in kDa (lane M). The red arrows indicate the recombinant OcCHI protein.

**Fig. S8** Western blot analysis of total protein isolated from *E. coli* expressing OcCHI (lane 1) and control empty vector (Lane CK).

**Fig. S9** Sequences alignment of *Oc4CLs*. The conserved putative AMP-binding motif (Box I) and the putative catalytic motif GEICIRG (Box II) is highlighted by red square. 12 amino acids proposed to function as a 4CL substrate specificity code are labelled with solid circles. The mutated amino acids between Oc4CL1 and Oc4CL6 are red shaded.

**Fig. S10** Sequences alignment of *OcCHS* proteins. Five conserved amino acids are labelled with solid circles. The mutated amino acid in OcCHS1 and OcCHS3 are red shaded.

Supplementary table captions

Table S1 Plasmids and strains used in this study

Table S2 Oligonucleotides used in this investigation

Table S3 Unigenes assigned to every step of (*2S*)-pinocembrin(**2**) biosynthetic pathway.

Table S4 ^1^H and ^13^C NMR data for the new fermentation product produced by strain 2 using *p*-coumaric acid (**6**) as the substrate (600 MHz for ^1^H NMR and 150 MHz for ^13^C NMR, D_2_O, J in Hz, *δ* in ppm)

| Pathway enzyme | Encoding sequences | |
| --- | --- | --- |
|  | Unigenes or contigs | Full-length cDNAs |
| 4CL-like | 19 | 7 |
| CHS-like | 3 | 3 |
| CHI-like | 4 | 1 |

Table 1

| Plasmid or strain | Relevant properties or genetic marker | Source or reference(s) |
| --- | --- | --- |
| Plasmids | | |
| pEASY^TM^-T1 | pUC ori, Amp^r^, Kan^r^ | TransGen |
| pCDFDuet-1 | CloDF13, Str^r^ | Novagen |
| pET-28a(+) | pBR22 ori, Kan^r^ | Novagen |
| pEASYOc4CL1 | pEASY^TM^-T1 derivative carrying *Oc4CL1* | This study |
| pEASYOc4CL2 | pEASY^TM^-T1 derivative carrying *Oc4CL2* | This study |
| pEASYOc4CL3 | pEASY^TM^-T1 derivative carrying *Oc4CL3* | This study |
| pEASYOc4CL4 | pEASY^TM^-T1 derivative carrying *Oc4CL4* | This study |
| pEASYOc4CL5 | pEASY^TM^-T1 derivative carrying *Oc4CL5* | This study |
| pEASYOc4CL6 | pEASY^TM^-T1 derivative carrying *Oc4CL6* | This study |
| pEASYOc4CL7 | pEASY^TM^-T1 derivative carrying *Oc4CL7* | This study |
| pEASYOcCHS1 | pEASY^TM^-T1 derivative carrying *OcCHS1* | This study |
| pEASYOcCHS2 | pEASY^TM^-T1 derivative carrying *OcCHS2* | This study |
| pEASYOcCHS3 | pEASY^TM^-T1 derivative carrying *OcCHS3* | This study |
| pEASYOcCHI | pEASY^TM^-T1 derivative carrying *OcCHI* | This study |
| pET28aOc4CL1 | pET-28a(+) derivative carrying *Oc4CL1* | This study |
| pET28aOc4CL2 | pET-28a(+) derivative carrying *Oc4CL2* | This study |
| pET28aOc4CL3 | pET-28a(+) derivative carrying *Oc4CL3* | This study |
| pET28aOc4CL4 | pET-28a(+) derivative carrying *Oc4CL4* | This study |
| pET28aOc4CL5 | pET-28a(+) derivative carrying *Oc4CL5* | This study |
| pET28aOc4CL6 | pET-28a(+) derivative carrying *Oc4CL6* | This study |
| pET28aOc4CL7 | pET-28a(+) derivative carrying *Oc4CL7* | This study |
| pET28aOcCHS1 | pET-28a(+) derivative carrying *OcCHS1* | This study |
| pET28aOcCHS2 | pET-28a(+) derivative carrying *OcCHS2* | This study |
| pET28aOcCHS3 | pET-28a(+) derivative carrying *OcCHS3* | This study |
| pET28aOcCHI | pET-28a(+) derivative carrying *OcCHI* | This study |
| pCDF-MSCHI | pCDFDuet-1 derivative carrying *MsCHI* | This study |
| pCDF-OcCHS2 | pCDFDuet-1 derivative carrying *OcCHS2* | This study |
| pCDF-OcCHS1-MsCHI | pCDFDuet-1 derivative carrying *OcCHS1* and *MsCHI* | This study |
| pCDF-OcCHS2-MsCHI | pCDFDuet-1 derivative carrying *OcCHS2* and *MsCHI* | This study |
| pCDF-OcCHS3-MsCHI | pCDFDuet-1 derivative carrying *OcCHS3* and *MsCHI* | This study |
| pCDF-OcCHS2-OcCHI | pCDFDuet-1 derivative carrying *OcCHS2* and *OcCHI* | This study |
| pET28a-Oc4CL1-OcCHS2 | pET-28a(+) derivative carrying *Oc4CL1* and *OcCHS2* | This study |
| pET28a-OptOc4CL1 | pET-28a(+) derivative carrying codon-optimized *Oc4CL1* | This study |
| pCDF-OptOcCHS2-MsCHI | pCDFDuet-1 derivative carrying codon-optimized *OcCHS2* and *MsCHI* | This study |
| pCDF-OptOcCHS2-OptMsCHI | pCDFDuet-1 derivative carrying codon-optimized *OcCHS2* and codon-optimized *MsCHI* | This study |
| Strains | | |
| Trans1-T1 | F- φ80 (*lacZ*)△M15 △*lac*X74 hsdR(r_k_^-^, m_k_^+^) △*rec*A1398 *end*Al *ton*A | TransGen |
| *Trans*etta(DE3) | F^-^ *omp*T *hsd*SB(r_B_^-^m_B_^-^) *gal* *dcm* *lac*Y1(DE3) pRARE(argU, argW, ilex, glyT, leuW, proL)(Cam^r^) | TransGen |
| Strain 1 | *Trans*etta(DE3) harboring pET28a-Oc4CL1 and pCDF-OcCHS1-MsCHI | This study |
| Strain 2 | *Trans*etta(DE3) harboring pET28a-Oc4CL1 and pCDF-OcCHS2-MsCHI | This study |
| Strain 3 | *Trans*etta(DE3) harboring pET28a-Oc4CL1 and pCDF-OcCHS3-MsCHI | This study |
| Strain 4 | *Trans*etta(DE3) harboring pET28a-Oc4CL1 and pCDF-OcCHS2 | This study |
| Strain 5 | *Trans*etta(DE3) harboring pET28a-Oc4CL1and pCDF-OcCHS2-OcCHI | This study |
| Strain 6 | *Trans*etta(DE3) harboring pET28a-Oc4CL1-OcCHS2 and pCDF-MsCHI | This study |
| Strain 7 | *Trans*etta(DE3) harboring pET28a-Oc4CL1 and pCDF-OptOcCHS2-MsCHI | This study |
| Strain8 | *Trans*etta(DE3) harboring pET28a-Oc4CL1 and pCDF-OptOcCHS2-OptMsCHI | This study |
| Strain9 | *Trans*etta(DE3) harboring pET28a-OptOc4CL1 and pCDF-OptOcCHS2-MsCHI | This study |
| Strain 10 | *Trans*etta(DE3) harboring pET28a-OptOc4CL1 and pCDF-OptOcCHS2-OptMsCHI | This study |
| Strain 11 | *Trans*etta(DE3) harboring pET28a-OptOc4CL1 and pCDF-OcCHS2-MsCHI | This study |

Table 2

| Position | The fermentation product produced by strain 2 using *p*-coumaric acid (**6**) as the substrate | | Reference | |
| --- | --- | --- | --- | --- |
|  | *δ*_H_ | *δ*_C_ | *δ*_H_ | *δ*_C_ |
| 2 | 5.34 dd (12.9, 3.0) | 80.54 | 5.35 dd (12.9, 2.9) | 80.5 |
| 3 | 3.11 dd (17.1, 12.9)  2.70 dd (17.1, 3.0) | 44.10 | 3.13 dd (17.1, 12.9)  2.71 dd (17.1, 2.9) | 44.0 |
| 4 |  | 197.83 |  | 197.8 |
| 5 |  | 165.52 |  | 165.52 |
| 6 | 5.90 d (2.2) | 97.07 | 5.91 d (2.1) | 97.1 |
| 7 |  | 168.40 |  | 168.4 |
| 8 | 5.88 d (2.2) | 96.19 | 5.92 d (2.1) | 96.2 |
| 9 |  | 165.52 |  | 165.5 |
| 10 |  | 116.36 |  | 113.4. |
| 1’ |  | 131.14 |  | 131.1 |
| 2’ | 7.31 d (8.7) | 129.07 | 7.33 d (8.5) | 129.1 |
| 3’ | 6.82 d (8.7) | 116.36 | 6.84 d (8.5) | 116.3 |
| 4’ |  | 159.08 |  | 159.0 |

Table 3

| Primer | Sequence (5’ to 3’) | Purpose |
| --- | --- | --- |
| FCL1-1 | TTAATTAGAAGCAAGCAAGC | Amplification of Oc4CL1 from *O.caudatum* |
| RCL1-1 | CTTTTCGACGGTCAGATCGAG | Amplification of Oc4CL1 from *O.caudatum* |
| FCL1-2 | ATGGGCTCCATCCCGTCGG | Amplification of Oc4CL1 from *O.caudatum* |
| RCL1-2 | TCACTGCTGAGGGCCGTTAG | Amplification of Oc4CL1 from *O.caudatum* |
| FCL2-1 | CTGCAAGCAATGGCGGCG | Amplification of Oc4CL2 from *O.caudatum* |
| RCL2-1 | GCATGCAATTGTATGATCGATTT | Amplification of Oc4CL2 from *O.caudatum* |
| FCL2-2 | ATGGCGGCGAAATCGTGCTAC | Amplification of Oc4CL2 from *O.caudatum* |
| RCL2-2 | CTACAGTTTTGATCGTCTCT | Amplification of Oc4CL2 from *O.caudatum* |
| FCL3-1 | ACAATTATGGAAGGCCTCAC | Amplification of Oc4CL3 from *O.caudatum* |
| RCL3-1 | AAAACTAAAGTCCAACATAG | Amplification of Oc4CL3 from *O.caudatum* |
| FCL3-2 | ATGGAAGGCCTCACCCTTAC | Amplification of Oc4CL3 from *O.caudatum* |
| RCL3-2 | CTAAGCTCCAGACTTGGGC | Amplification of Oc4CL3 from *O.caudatum* |
| FCL4-1 | CTTCTTTAAGAAAAGGAGTG | Amplification of Oc4CL4 from *O.caudatum* |
| RCL4-1 | AACTCTCATCAACAGAAGAAC | Amplification of Oc4CL4 from *O.caudatum* |
| FCL4-2 | ATGGGAGAGGAGAGGGAGAG | Amplification of Oc4CL4 from *O.caudatum* |
| RCL4-2 | TTATAACCTGCTATACTTCAC | Amplification of Oc4CL4 from *O.caudatum* |
| FCL5-1 | CAGTGAGAGCGAGGAGAATC | Amplification of Oc4CL5 from *O.caudatum* |
| RCL5-1 | CGACAAAGGTACATCTATTC | Amplification of Oc4CL5 from *O.caudatum* |
| FCL5-2 | ATGGAGGGCACACTCCGTTG | Amplification of Oc4CL5 from *O.caudatum* |
| RCL5-2 | TCAGAGCTTGCTGAAAAGGC | Amplification of Oc4CL5 from *O.caudatum* |
| FCL6-1 | ATATCGTTAATTTTCCGGCC | Amplification of Oc4CL6 from *O.caudatum* |
| RCL6-1 | TCATGGCAAATGATGGAAAT | Amplification of Oc4CL6 from *O.caudatum* |
| FCL6-2 | ATGATCACCGTCGCCGCG | Amplification of Oc4CL6 from *O.caudatum* |
| RCL6-2 | TCAAGAGTCATTAAGAAAAG | Amplification of Oc4CL6 from *O.caudatum* |
| FCL8-1 | GACAACTCCGTAGCTAAGCG | Amplification of Oc4CL7 from *O.caudatum* |
| RCL8-1 | TCAGACTTAGCCGTGATGCC | Amplification of Oc4CL7 from *O.caudatum* |
| FCL8-2 | ATGGAAGGCACAGTCCGTAG | Amplification of Oc4CL7 from *O.caudatum* |
| RCL8-2 | TCAAAGCCTACTAGAAATAAT | Amplification of Oc4CL7 from *O.caudatum* |
| Fchas1-1 | CATGGATGCCATTGCAAGC | Amplification of OcCHS1 from *O.caudatum* |
| Fchas1-2 | ATGATCCCCATCTCCGAGAG | Amplification of OcCHS1 from *O.caudatum* |
| Rchas1-1 | TTCCCACATGCACATAAGCT | Amplification of OcCHS1 from *O.caudatum* |
| Rchas1-2 | TCACGCGCAGCGGAGGACG | Amplification of OcCHS1 from *O.caudatum* |
| Fchas2-1 | CATCTCAAATGGCGCCCAAT | Amplification of OcCHS2 from *O.caudatum* |
| Fchas2-2 | ATGAACATTGAGGAGATCAG | Amplification of OcCHS2 from *O.caudatum* |
| Rchas2-1 | GTATACGGACGATCAGCTAG | Amplification of OcCHS2 from *O.caudatum* |
| Rchas2-2 | CTAGCCAGCGAAGGGCACG | Amplification of OcCHS2 from *O.caudatum* |
| Fchas3-1 | GCTAACTCTAATATTTTTAAGGC | Amplification of OcCHS3 from *O.caudatum* |
| Fchas3-2 | ATGGAGTCCATTGCTTATG | Amplification of OcCHS3 from *O.caudatum* |
| Rchas3-1 | TTCCCACATGCACATAAGCT | Amplification of OcCHS3 from *O.caudatum* |
| Rchas3-2 | TCACGCGCAGCGGAGGACG | Amplification of OcCHS3 from *O.caudatum* |
| FCHI1-1 | CTTAATCACTTCTCTCCTTCAC | Amplification of OcCHI from *O.caudatum* |
| RCHI1-1 | GAGAGAGAGAGAAAACACTCT | Amplification of OcCHI from *O.caudatum* |
| FCHI1-2 | ATGGGATCTGAGATGGTGATG | Amplification of OcCHI from *O.caudatum* |
| RCHI1-2: | TTAAGCTGAAGCCAGAATGGT | Amplification of OcCHI from *O.caudatum* |
| FInfCL1 | TCGCGGATCCGAATTCATGGGCTCCATCCCGTCGG | Construction of pET28a-Oc4CL1 |
| RInfCL1 | GTGCGGCCGCAAGCTTTCACTGCTGAGGGCCGTTAG | Construction of pET28a-Oc4CL1 |
| FInfCL2 | TCGCGGATCCGAATTCATGGCGGCGAAATCGTGCTAC | Construction of pET28a-Oc4CL2 |
| RInfCL2 | GTGCGGCCGCAAGCTTCTACAGTTTTGATCGTCTCT | Construction of pET28a-Oc4CL2 |
| FInfCL3 | TCGCGGATCCGAATTCATGGAAGGCCTCACCCTTAC | Construction of pET28a-Oc4CL3 |
| RInfCL3 | GTGCGGCCGCAAGCTTCTAAGCTCCAGACTTGGGC | Construction of pET28a-Oc4CL3 |
| FInfCL4 | TCGCGGATCCGAATTCATGGGAGAGGAGAGGGAGAG | Construction of pET28a-Oc4CL4 |
| RInfCL4 | GTGCGGCCGCAAGCTTTTATAACCTGCTATACTTCAC | Construction of pET28a-Oc4CL4 |
| FInfCL5 | TCGCGGATCCGAATTCATGGAGGGCACACTCCGTTG | Construction of pET28a-Oc4CL5 |
| RInfCL5 | GTGCGGCCGCAAGCTTTCAGAGCTTGCTGAAAAGGC | Construction of pET28a-Oc4CL5 |
| FInfCL6 | TCGCGGATCCGAATTCATGATCACCGTCGCCGCG | Construction of pET28a-Oc4CL6 |
| RInfCL6 | GTGCGGCCGCAAGCTTTCAAGAGTCATTAAGAAAAG | Construction of pET28a-Oc4CL6 |
| FInfCL7 | TCGCGGATCCGAATTCATGGAAGGCACAGTCCGTAG | Construction of pET28a-Oc4CL7 |
| RInfCL7 | GTGCGGCCGCAAGCTTTCAAAGCCTACTAGAAATAAT | Construction of pET28a-Oc4CL7 |
| F28achas1 | AATGGGTCGCGGATCC ATGATCCCCATCTCCGAGAG | Construction of pET28a-OcCHS1 |
| R28achas1 | GTGCGGCCGCAAGCTT TCACGCGCAGCGGAGGACG | Construction of pET28a-OcCHS1 |
| F28achas2 | AATGGGTCGCGGATCC ATGAACATTGAGGAGATCAG | Construction of pET28a-OcCHS2 |
| R28achas2 | GTGCGGCCGCAAGCTT CTAGCCAGCGAAGGGCACG | Construction of pET28a-OcCHS2 |
| F28achas3 | AATGGGTCGCGGATCC ATGGAGTCCATTGCTTATG | Construction of pET28a-OcCHS3 |
| R28achas3 | GTGCGGCCGCAAGCTT TCACGCGCAGCGGAGGACG | Construction of pET28a-OcCHS3 |
| F28aCHI | AATGGGTCGCGGATCCATGGGATCTGAGATGGTGATG | Construction of pET28a-OcCHI |
| R28aCHI | GTGCGGCCGCAAGCTTTTAAGCTGAAGCCAGAATGG | Construction of pET28a-OcCHI |
| Fpcdfmschi | ACCACAGCCAGGATCCATGGCAGCAAGCATT | Construction of pCDF-MsCHI |
| Rpcdfmschi | ATGCGGCCGCAAGCTTTCAGTTACCGATTTTA | Construction of pCDF-MsCHI |
| Fpcdfchs1 | AAGGAGATATACATATGATGATCCCCATCTCC | Construction of pCDF-OcCHS1-MsCHI |
| Rpcdfchs1 | CTTTACCAGACTCGAGTCACGCGCAGCGGAG | Construction of pCDF-OcCHS1-MsCHI |
| Fpcdfchs2 | AAGGAGATATACATATGATGAACATTGAGGAG | Construction of pCDF-OcCHS2-MsCHI |
| Rpcdfchs2 | CTTTACCAGACTCGAGCTAGCCAGCGAAGGG | Construction of pCDF-OcCHS2-MsCHI |
| Fpcdfchs3 | AAGGAGATATACATATGATGGAGTCCATTGCT | Construction of pCDF-OcCHS3-MsCHI |
| Rpcdfchs3 | CTTTACCAGACTCGAGTCACGCGCAGCGGAG | Construction of pCDF-OcCHS3-MsCHI |
| FpcdfCHI | ACCACAGCCAGGATCCATGGGATCTG | Construction of pCDF-OcCHS2-OcCHI |
| RpcdfCHI | ATGCGGCCGCAAGCTTTTAAGCTGAA | Construction of pCDF-OcCHS2-OcCHI |
| F4cl1chs | AGGAATGGTGCATGCTAGAGGATCGAGATCTCG | Construction of pET28a-Oc4CL1-OcCHS2 |
| R4cl1chs | ATTTCGCGGGATCGAGATCTATCCGGATATAGTTC | Construction of pET28a-Oc4CL1-OcCHS2 |
| FY284cl1 | TCGCGGATCCGAATTCATGGGTTCTATCCCG | Construction of pET28a-OptOc4CL1 |
| RY284cl1 | GTGCGGCCGCAAGCTTTTACTGCTGCGGACC | Construction of pET28a-OptOc4CL1 |
| FYcdfchs2 | AAGGAGATATACATATGATGAACATCGAAGAA | Construction of pCDFOptOcCHS2 and pCDF-OptCHS2-OptMsCHI |
| RYcdfchs2 | CTTTACCAGACTCGAGTTAACCAGCGAACGG | Construction of pCDFOptOcCHS2 and pCDF-OptCHS2-OptMsCHI |
| FYpcdfmschi | ACCACAGCCAGGATCCATGGCAGCAAGCATT | Construction of pCDF-optMsCHI an pCDF-OcCHS2-OptMSI |
| RYpcdfmschi | ATGCGGCCGCAAGCTTTTAGTTACCGATTTT | Construction of pCDF-optMsCHI an pCDF-OcCHS2-OptMSI |

Table 4

References

1. Prescott, A. G., Stamford N. P. J., Wheeler G. Firmin J. L. *In vitro* properties of a recombinant flavonol synthase from *Arabidopsis thaliana*, Phytochemistry. 2002; 60:589-93.
