# Supplementary material for: Transcriptome-enabled discovery and functional characterization of enzymes related to (2S)-pinocembrin biosynthesis from Ornithogalum caudatum and their application for metabolic engineering
Source: Microb Cell Fact. 2016 Feb 4;15:27. doi: 10.1186/s12934-016-0424-8 (PMC4743118; doi:10.1186/s12934-016-0424-8)
Supplement: Supplementary file 6 — 10.1186/s12934-016-0424-8 HPLC analysis of the reaction products of recombinant OcCHS2 protein. A, HPLC analysis of the reaction product from E.coli[pET28a]; B, HPLC analysis of the reaction products of recombinant Oc4CL1 protein using trans-cinnamic acid (5) as the substrate; C, HPLC analysis of the reaction products of recombinant OcCHS2 protein using reaction products of recombinant Oc4CL1 as the substrate. peak 1, trans-cinnamic acid (5); peak 2, trans-cinnamoyl CoA (10), peak 3, pinocembrin chalcone (4). [file 12934_2016_424_MOESM5_ESM.doc]

Fig. S4
