# Supplementary material for: Transcriptome-enabled discovery and functional characterization of enzymes related to (2S)-pinocembrin biosynthesis from Ornithogalum caudatum and their application for metabolic engineering
Source: Microb Cell Fact. 2016 Feb 4;15:27. doi: 10.1186/s12934-016-0424-8 (PMC4743118; doi:10.1186/s12934-016-0424-8)
Supplement: Supplementary file 7 — 10.1186/s12934-016-0424-8 HPLC analysis of the fermentation products of strains 1-3. A, HPLC analysis of the fermentation products of strain E. coli[pET28a] using p-coumaric acid (6) as the substrate; B, HPLC analysis of the fermentation products of strains 1 using p-coumaric acid (6) as the substrate; C, HPLC analysis of the fermentation products of strains 2 using p-coumaric acid (6) as the substrate; D, HPLC analysis of the fermentation products of strains 3 using p-coumaric acid (6) as the substrate; peak 1, p-coumaric acid (6); peak 2, naringenin; The inserted tablet represented the UV absorbance of the product naringenin. [file 12934_2016_424_MOESM6_ESM.doc]

Fig.S5
