# Supplementary material for: Transcriptome-enabled discovery and functional characterization of enzymes related to (2S)-pinocembrin biosynthesis from Ornithogalum caudatum and their application for metabolic engineering
Source: Microb Cell Fact. 2016 Feb 4;15:27. doi: 10.1186/s12934-016-0424-8 (PMC4743118; doi:10.1186/s12934-016-0424-8)
Supplement: Supplementary file 8 — 10.1186/s12934-016-0424-8 SDS-PAGE analysis of total proteins stained with silver nitrate. Lane 1, total protein from E. coli expressing OcCHI; lane CK, total protein from bacteria containing the empty vector alone; Molecular masses of markers are shown to the left in kDa (lane M). The red arrows indicate the recombinant OcCHI protein. [file 12934_2016_424_MOESM7_ESM.doc]

Fig.S6
