# Supplementary material for: Transcriptome-enabled discovery and functional characterization of enzymes related to (2S)-pinocembrin biosynthesis from Ornithogalum caudatum and their application for metabolic engineering
Source: Microb Cell Fact. 2016 Feb 4;15:27. doi: 10.1186/s12934-016-0424-8 (PMC4743118; doi:10.1186/s12934-016-0424-8)
Supplement: Supplementary file 9 — 10.1186/s12934-016-0424-8 Western blot analysis of total protein isolated from E. coli expressing OcCHI (lane 1) and control empty vector (Lane CK). [file 12934_2016_424_MOESM8_ESM.doc]

Fig. S7
