# Supplementary material for: Transcriptome-enabled discovery and functional characterization of enzymes related to (2S)-pinocembrin biosynthesis from Ornithogalum caudatum and their application for metabolic engineering
Source: Microb Cell Fact. 2016 Feb 4;15:27. doi: 10.1186/s12934-016-0424-8 (PMC4743118; doi:10.1186/s12934-016-0424-8)
Supplement: Supplementary file 10 — 10.1186/s12934-016-0424-8 Sequences alignment of Oc4CLs. The conserved putative AMP-binding motif (Box I) and the putative catalytic motif GEICIRG (Box II) is highlighted by red square. 12 amino acids proposed to function as a 4CL substrate specificity code are labelled with solid circles. The mutated amino acids between Oc4CL1 and Oc4CL6 are red shaded. [file 12934_2016_424_MOESM9_ESM.doc]

Fig. S8
