# Supplementary material for: Transcriptome-enabled discovery and functional characterization of enzymes related to (2S)-pinocembrin biosynthesis from Ornithogalum caudatum and their application for metabolic engineering
Source: Microb Cell Fact. 2016 Feb 4;15:27. doi: 10.1186/s12934-016-0424-8 (PMC4743118; doi:10.1186/s12934-016-0424-8)
Supplement: Supplementary file 11 — 10.1186/s12934-016-0424-8 Sequences alignment of OcCHS proteins. Five conserved amino acids are labelled with solid circles. The mutated amino acid in OcCHS1 and OcCHS3 are red shaded. [file 12934_2016_424_MOESM10_ESM.doc]

Fig. S9
